# Boronium Ionic Liquids for High-Voltage Supercapacitors

**DOI:** 10.1021/acsaenm.5c00888

**Published:** 2025-11-24

**Authors:** Whirang Cho, Christopher D. Stachurski, Zachary G. Neale, Miaomiao Ma, Margaret E. Crowley, Matthias Zeller, James H. Davis, Paul C. Trulove, David P. Durkin

**Affiliations:** † Department of Chemistry, 32722 U.S. Naval Academy, Annapolis, Maryland 21402, United States; ‡ Surface Chemistry Branch, U.S. Naval Research Laboratory, Washington, District of Columbia 20375, United States; § Carderock Division, 97071 Naval Surface Warfare Center, Bethesda, Maryland 20817, United States; ∥ Department of Chemistry, 5557 University of South Alabama, Mobile, Alabama 36688, United States; ⊥ Department of Chemistry, 8522 Purdue University, West Lafayette, Indiana 47907, United States

**Keywords:** boronium, ionic liquid (ILs), supercapacitor, carbon nanofoam, high power density

## Abstract

Boronium ionic liquids (BILs) are an emergent class of electrolytes with high electrochemical stability afforded by charge delocalization across the cation. BILs are of particular interest for electrochemical energy storage (EES) devices because of their large voltage window. Here, a series of BILs were systematically evaluated as electrolytes in symmetric double-layer capacitors equipped with carbon nanofoam paper (CNFP) architected electrodes. First, the operational voltage window and capacitive properties of supercapacitor cells composed of BILs and CNFP electrodes were evaluated in a two-electrode configuration by using cyclic voltammetry (CV). Then, galvanostatic charge–discharge (GCD) cycling was used to assess the capacitance, energy density, power density, and long-term stability of cells assembled with the BIL electrolyte. Our results show excellent capacitive behavior of the cells assembled with a series of ammonium-, imidazolium-, and pyrrolidinium-based BILs, with nearly rectangular CV curves across a range of scan rates. Specifically, the methylpyrrolidinium-substituted BIL electrolyte ([(1-m-pyrr)­N_111_BH_2_]­TFSI, TFSI: bis­(trifluoromethane)­sulfonimide) presents higher ionic conductivity (1.82 mS cm^–1^ at 25 °C) compared to other BIL analogues and a wide operating voltage window of ∼3.7 V. These properties of [(1-m-pyrr)­N_111_BH_2_]­TFSI deliver an appreciable energy density of 16.3 Wh kg^–1^ (at a power density of 36.4 W kg^–1^), whereas [(1-a-pyrr)­N_111_BH_2_]­TFSI achieves a maximum power density of 13.9 kW kg^–1^. Overall, these BILs display excellent power density and sufficient energy density with the advantage of steadily delivering the energy at high power density. High cycling durability is also possible with the BILs supercapacitor cells, which maintain a capacitance retention above 90% after undergoing 1000 charge–discharge cycles at a current density of 0.5 A g^–1^. Finally, the specific capacitance, energy density, and power density of ammonium- and pyrrolidinium-based BILs exhibit a delicate dependence on temperature intended to facilitate the diffusion kinetics of BILs, confirming thermal resilience with no additional performance advantage.

## Graphical abstract

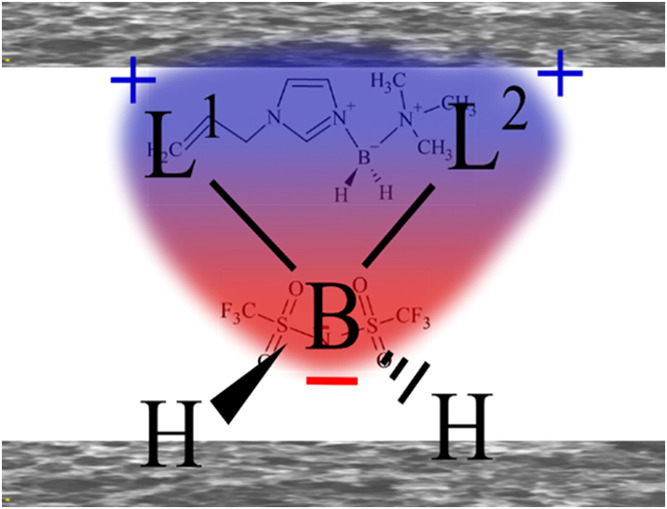

## Introduction

Society’s insatiable demand for electric power is driving research efforts to advance energy storage and conversion technologies. Of the various electrochemical energy storage (EES) systems, supercapacitors have broad application in multiple sectors, including automotive, consumer electronics, aerospace and defense, due to their rapid charge–discharge times, high power density, and stability.^1–5^ Supercapacitors are able to bridge the gap between traditional high-power capacitors and high-energy batteries, while maintaining higher energy densities than electrolytic capacitors.^6–8^ Beyond providing swift bursts of energy, solid-state supercapacitors can be thinner and effectively integrated into flexible devices, making them attractive power management systems for wearable electronics.^9,10^ Electrolytes play a significant role in EES devices by providing electronic insulation between two electrodes while facilitating ionic conduction.^11,12^ Since energy density follows a quadratic dependence on cell voltage, enlarging the electrochemical potential window of the electrolyte is imperative to realizing enhanced device performance. However, conventional organic and aqueous electrolytes present several limitations: organic electrolytes often exhibit flammability, intrinsic toxicity, and volatility, while aqueous electrolytes have limited electrochemical stability. These limitations can compromise the efficiency of cell performance, thus highlighting an urgent need to develop better high-voltage electrolytes that can improve device capacitance, energy, and power density.

Room-temperature ionic liquids (RTILs) are ionic compounds featuring a broad liquid temperature range that typically melt below 100 °C.^13,14^ RTILs are outstanding candidates for supercapacitor electrolytes because they have high ionic conductivity, low vapor pressure, high thermal stability, and superior electrochemical stability windows compared to traditional aqueous or organic electrolytes affording higher charge accumulation.^3,15–21^ In addition, the ion transport and dynamics properties of many RTILs have been shown to have positive effects on the electrolyte/electrode interface when used for EES.^22,23^ Recently, boronium ionic liquids (BILs) have emerged as promising EES candidates due to their high electrochemical and thermal stability, high-voltage windows, and the tailorable nature of the boronium cation.^24–26^ BILs were pioneered by Davis and co-workers, who introduced many novel compounds with cations in the form [L^1^L^2^-BH_2_]^+^, where L^1^and L^2^ are Lewis basic organic ligands.^27^ Rüther et al., evaluated some of these BILs (with TFSI) as electrolytes for lithium batteries, specifically [(1-methylimidazolium)_2_BH_2_]^+^, [(1-butylimidazolium)_2_BH_2_]^+^, and [(N_111_N_112_)­BH_2_]^+^ (N_111_ = trimethylamine; N_112_ = dimethylethylamine).^24^ Stachurski et al. showed that BILs containing pyrrolidinium ligands can have enhanced electrochemical stability windows and higher thermal stability.^28^ In a follow-on study, Stachurski et al. reported the physical and chemical properties of vinyl and allyl-substituted poly­(BILs), which have potential to be incorporated into solid or pseudosolid state supercapacitor devices.^29^ The adoption of an asymmetric functionalization strategy offers significant advantages, such as weaker ionic coordination between cations and anions, and more rapid charge development on the electrode surfaces.^30^

This work expands upon these previous studies, leveraging the BILs’ electrochemical and physicochemical characteristics to explore their potential as effective electrolytes for supercapacitor applications. We investigate a series of ammonium-, imidazolium-, and pyrrolidinium-based BILs and poly­(BILs) to determine their (i) operating voltage windows, (ii) specific capacitance, (iii) energy density, (iv) power density, and (v) long-term stability. Carbon nanofoam paper (CNFP) serves as the electrode system, within which the BILs are evaluated. Porous carbons are extensively used as electrodes in supercapacitors because their highly accessible surface area enhances capacitance and consequently improves cell performance. Already proven in other EES systems, freestanding CNFP electrodes offer a disordered 3D network of mesopores and/or macropores, high specific surface area (ca. 553 m^2^ g_Total_^–1^), and internal free volume to sustain ion balance during high-rate charge–discharge.^31,32^ When high-voltage BIL electrolytes and high-surface-area CNFPs are combined as supercapacitor cells, they deliver high power density, competitive capacitance and energy density, and long-term stability across all test conditions (including elevated temperatures of 40 and 50 °C) compared to traditional ILs.

## Experimental Section

### Materials

Tetrabutylammonium hexafluorophosphate ([TBA]­PF_6_, 98%) was obtained from Sigma-Aldrich and used without further purification. Acetonitrile (ACN, UV/HPLC grade) was obtained from Pharmco and dried beforehand by using 3 Å molecular sieves. Single-ply carbon nanofoam papers (CNFPs) were synthesized via a resorcinol–formaldehyde (RF) sol–gel process followed by pyrolysis (specific surface area: ca. 553 m^2^ g^–1^) and used as electrodes.^31,33^ X-ray photoelectron spectroscopy (XPS) reveals that the surface (ca. < 10 nm) of the CNFP electrode primarily consists of carbon (92.2 atom %), oxygen (5.4 atom %), and nitrogen (1.3 atom %), as expected from prior reports.^33–37^ CNFPs and commercial glass microfiber filter papers (Whatman glass microfiber grade GF/A, 1.6 μm pore size) were carefully punched into 12 mm diameter circles with a hollow-punch tool.

### Synthesis of Electrolytes

In this study, four different BIL cations (Figure 1) were synthesized in accordance with published methods, paired with the bis­(trifluoromethanesulfonyl)­imide (TFSI) anion, and tested as electrolytes in supercapacitor cells.^28,29^ The physical, thermal, and electrochemical properties of the BILs strongly depend on their structure, with exceptional intrinsic chemical stability afforded by the fully coordinated boron center.^26,38^ Each cation structure was verified by ^1^H NMR and single crystal X-ray diffractometry (XRD); thermal stability was evaluated by thermogravimetric analysis (TGA) and differential scanning calorimetry (DSC).^39,40^ The details of the synthesis and characterization of BILs a-c in Figure 1 are given in our previous work.^28,29^ The synthesis of [(1-a-imid)­N_111_BH_2_]­TFSI is adapted from previously published methods and is described in the Supporting Information. Characterization of this new BIL is shown in Figures S1–S3.

**Figure 1:**
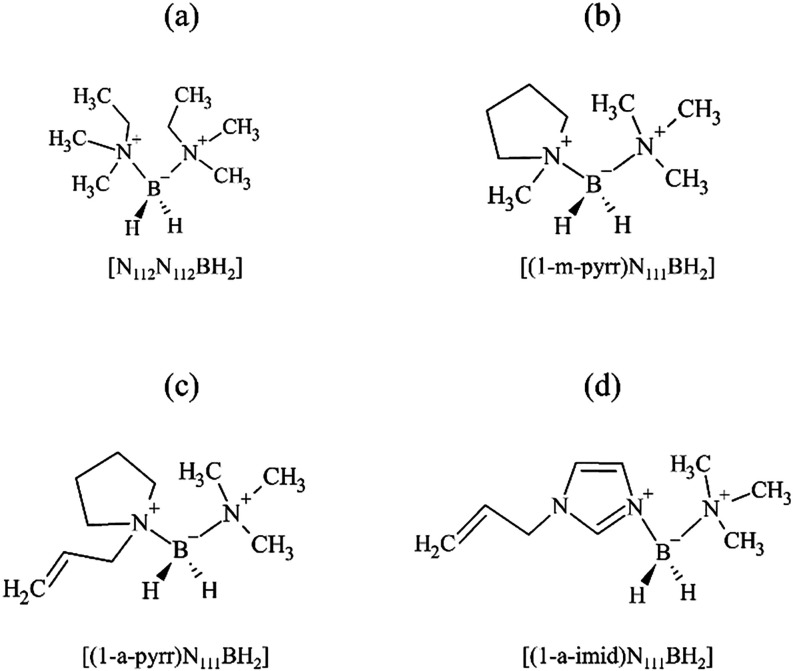
Structure of the boronium ionic liquid (BIL) cations investigated in this study. (a) Bis­(dimethylethylamine)­boronium, (b) (1-methylpyrrolidinium)­boronium, (c) (1-allylpyrrolidinium)­boronium, and (d) (1-allylimidazolium)­boronium. After synthesis, all iodide cations were anion-exchanged to contain the common anion bis­(trifluoromethane)­sulfonimide [TFSI].

### Electrochemical Measurements

Conductivity measurements were performed using a Biologic MCS-10 instrument on individual cells consisting of two carbon-coated platinum parallel plate electrodes with 1 cm spacing. The system was calibrated with a 0.01 M KCl standard solution prior to analyzing any BIL electrolyte.^29,41^ Each cell was calibrated between 25 and 50 °C in 5 °C increments, and the cell constant at each temperature was factored into the average value for each set of electrodes. Each supercapacitor testing cell was sandwiched with two 12 mm diameter carbon nanofoam paper (CNFP) disc electrodes, two layers of 12 mm diameter glass microfiber filter separators, and a gold-plated spring (spring constant: 32.6 N/m) and then assembled into a TSC battery cell (RHD instruments) (Figure S4). The PEEK casing of the battery cell hermetically sealed the cell. Each electrode and separator were dried under vacuum (60 °C) for 2 days and then impregnated with BIL electrolyte for 2 days before being tested. The supercapacitor cells were assembled and tested entirely in a nitrogen-filled glovebox (<1 ppm of H_2_O). At least three supercapacitor cells were tested for each system using a fresh electrolyte and CNFP electrodes under the same conditions. Cyclic voltammetry (CV), galvanostatic charge–discharge (GCD), and electrochemical impedance spectroscopy (EIS) measurements were performed by using a Biologic SP-200 potentiostat. CV measurements were conducted with sequentially increasing voltage windows ranging from 0–2 V up to 0–4 V at a constant sweep rate of 20 mV s^–1^ to determine the operating voltage windows and stability of each electrolyte (Coulombic efficiency threshold ≥ 95%).^42,43^ Afterward, scan rates were varied from 5–200 mV s^–1^ at a cutoff potential ranging from 2.9 to 3.6 V for each BIL electrolyte. The charge–discharge data were then collected at the corresponding cutoff potential with varying current density from 0.05 to 1.5 A g^–1^, where g is the total active mass of the two electrodes. The cycling performance of each supercapacitor cell was evaluated for up to 1000 continuous charge–discharge cycles, and their specific capacitance (*C*_sp_) was determined by eq 1:

1 $$C_{\mathrm{sp}}=\frac{I_{m}\Delta t}{\Delta V}$$

where *I*_m_ is current density, Δ*t* is discharge time, and Δ*V* is the potential window between electrodes during the charge–discharge process. The cycling stability was evaluated through a minimum of 1000 charge–discharge cycles by eq 2:

2 $$\mathrm{capacitance}\;\mathrm{retention}=\frac{C_{n\mathrm{th}\;\mathrm{cycle}}}{C_{\max}}\times 100$$

where *C*_*n*th cycle_ is the capacitance calculated for the *n*th cycle and *C*_max_ is the maximum capacitance. The energy density (*E*, Wh kg^–1^) and power density (*P*, W kg^–1^) were then calculated by eqs 3 and 4:

3 $$E=\frac{1}{2}CV^{2}$$

4 $$P=\frac{3600E}{\Delta t}$$

where *V* is the operating voltage window and Δ*t* is the discharge time (s).

EIS spectra were obtained over the range of 1 MHz to 0.1 Hz at the cell’s open-circuit potential using an AC current amplitude of 10 mV. The thermal resilience of supercapacitor cells containing the ammonium- and pyrrolidinium-based BILs was evaluated by collecting the same electrochemical data (in triplicate) at 25 °C, 40 °C, and 50 °C in a custom-built heating block. Cell temperature was controlled and measured by using a Barnant 689–0000 temperature controller. Before each CV and GCD measurement, the cell was stabilized at each temperature for 2 h. Stabilization time was determined by conducting EIS measurements at the desired temperature, until the resistance of the cell remained constant.

## Results and Discussion

The ionic conductivity of each BIL was measured between 298 and 323 at 5 K steps (Figure S5). The fittings of these data indicate Arrhenius-like behavior of each electrolyte within the tested temperature range. At all measured temperatures, [(1-m-pyrr)­N_111_BH_2_]­TFSI consistently shows higher ionic conductivity (1.82 mS cm^–1^ at 25 °C) than the other three BILs. While still generally high, the ionic conductivity of [(1-a-pyrr)­N_111_BH_2_]­TFSI (0.73 mS cm^–1^ at 25 °C) is lower than that of [N_112_N_112_BH_2_]­TFSI (1.36 mS cm^–1^ at 25 °C), most likely because it has larger ligands and is slightly more viscous. Interestingly, the *T*_g_ of [(1-m-pyrr)­N_111_BH_2_]­TFSI (ca. −72 °C) is significantly higher than that of the [N_112_N_112_BH_2_]­TFSI (ca. −90 °C),^28^ suggesting the (1-m-pyrr) ligand (in [(1-m-pyrr)­N_111_BH_2_]^+^) is larger than the N_112_ (in [N_112_N_112_BH_2_]^+^). The enhanced ion mobility of [(1-m-pyrr)­N_111_BH_2_]­TFSI, despite its larger ligand structure,^28^ is likely due to the fact that it is less viscous. Reducing viscosity can directly improve electrochemical processes, such as charge transport, ionic conduction, and ion diffusion. Greater charge delocalization over the [(1-m-pyrr)­N_111_BH_2_] cation can also facilitate some dissociation from the TFSI anion,^44^ thereby enhancing its ion diffusion and charge transport. The appreciably high ion conductivity of [(1-a-imid)­N_111_BH_2_]­TFSI (1.50 mS cm^–1^ at 25 °C) also likely arises from a combination of its relative fluidity and charge delocalization presented by the polarizable allylimidazolium ligand.^45^

The impedance response of each BIL was measured by using EIS (Figure 2). A typical Nyquist plot shows a semicircle at the higher frequency region and a linear variation at the lower frequency domain.^46^ The vertical lines almost parallel to the Im­(z) axis of each BIL electrolyte indicate the capacitive behavior of all BILs under investigation. The high-frequency intercept on the real axis of the Nyquist plot is attributed to the bulk electrolyte resistance of the cells (*R*_s_). The measured *R*_s_ for [N_112_N_112_BH_2_]­TFSI (25 Ω), [(1-m-pyrr)­N_111_BH_2_]­TFSI (29 Ω), and [(1-a-imid)­N_111_BH_2_]­TFSI (29 Ω) are lower than the 48 Ω observed for [(1-a-pyrr)­N_111_BH_2_]­TFSI. This correlates with the lower ionic conductivity of the [(1-a-pyrr)­N_111_BH_2_]­TFSI electrolyte compared to the other three BIL electrolytes. The diameter of each semicircle in the Nyquist plots can be attributed to the interfacial charge-transfer resistance (*R*_ct_) arising from charge accumulation between the electrolyte and the surface of the electrode.^47^ The *R*_ct_ value of [(1-a-pyrr)­N_111_BH_2_]­TFSI (∼35 Ω) is lower compared to other BIL analogues, suggesting reduced ionic resistance from charge buildup at the electrolyte-electrode interface. This characteristic is favorable for the formation of an electric double layer.

**Figure 2:**
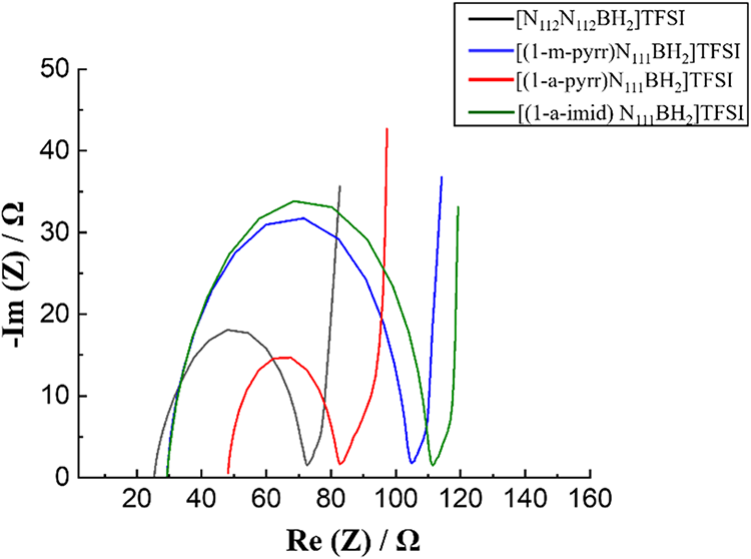
Nyquist plots of supercapacitor cells assembled with carbon nanofoam paper (CNFP) electrodes and each BIL electrolyte. Electrochemical impedance spectroscopy (EIS) was conducted at open-circuit potential with an applied AC voltage of 10 mV from 10^6^ to 10^–1^ Hz.

To determine the operating voltages of each electrolyte, CV measurements were performed with a sequentially increasing voltage window ranging from 0–2 V up to 0–4 V at a constant sweep rate of 20 mV s^–1^ (Figure 3). All four supercapacitor cells assembled with BILs exhibit nearly rectangular CV profiles, indicating good capacitive behavior (Figures 3a and S6). When a voltage higher than 3.6 V is applied to the cell, the inflection point and polarization current of [(1-m-pyrr)­N_111_BH_2_]­TFSI become more pronounced. While an ideal electric double-layer capacitor (EDLC) would exhibit a perfectly rectangular CV curve, in practice, the curves often deviate from this ideal shape. Deviation from an ideal rectangular CV curve in our system can be attributed to the intrinsic internal resistance of ionic liquids and the porosity of CNFP electrodes.^48,49^ [(1-m-pyrr)­N_111_BH_2_]­TFSI shows a slightly larger CV-enclosed area than the other BILs, indicating that it has a larger specific capacitance.

**Figure 3:**
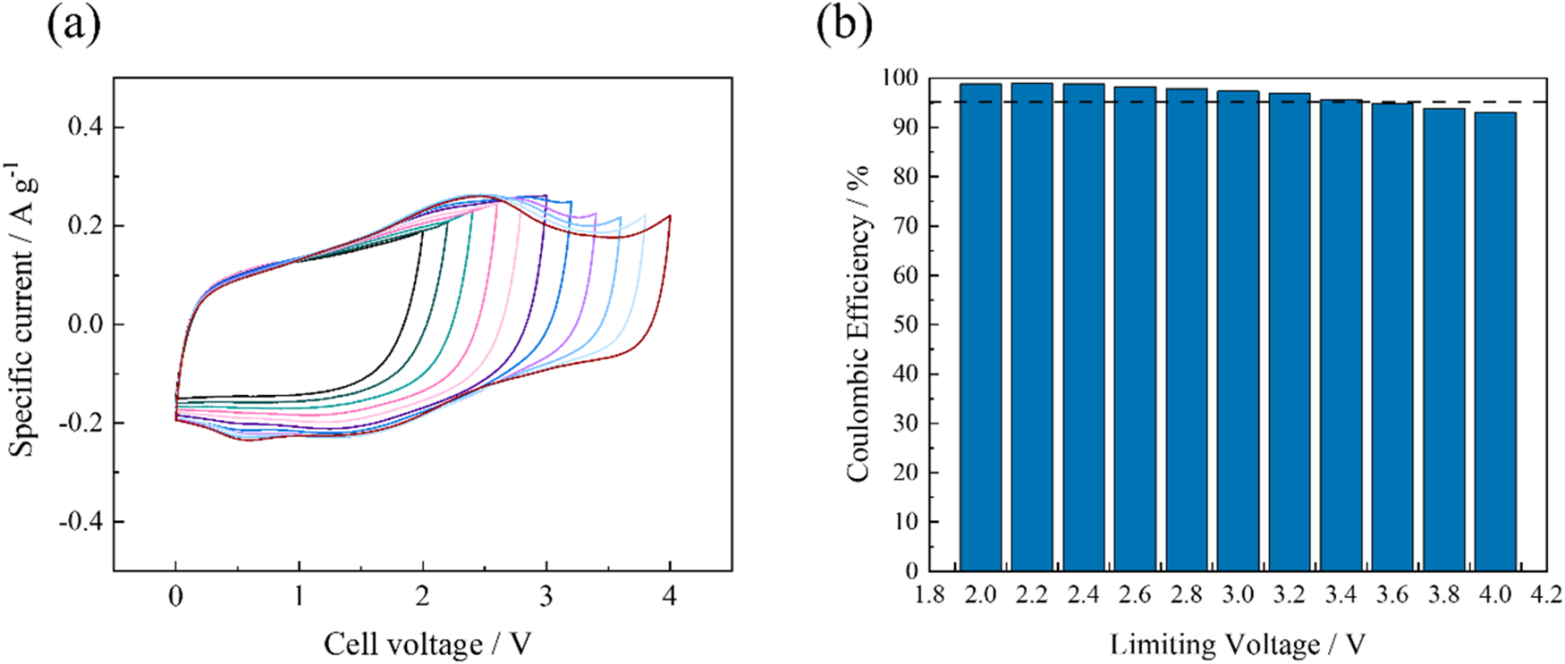
(a) Cyclic voltammetry (CV) curves of [(1-m-pyrr)­N_111_BH_2_]­[TFSI] within different voltage windows ranging from 0–2 to 0–4 V at a scan rate of 20 mV s^–1^. (b) Coulombic efficiency of [(1-m-pyrr)­N_111_BH_2_]­[TFSI] at different applied voltages. Coulombic efficiency was calculated from the ratio between the amount of charge and discharge at each applied voltage.

Coulombic efficiency is calculated from the ratio of the amount of discharge capacity to charge capacity from eq 5:

5 $$\mathrm{Coulombic}\;\mathrm{efficiency}=\frac{Q_{\mathrm{discharge}}}{Q_{\mathrm{charge}}}\times 100$$

The Coulombic efficiency of [(1-m-pyrr)­N_111_BH_2_]­TFSI gradually decreases as the applied voltage is increased (Figure 3b). A wider voltage window offers higher energy density; however, the cell must operate within a voltage window to prevent electrolyte degradation. To ensure the electrochemical integrity of the electrolyte, an appropriate operating voltage was determined experimentally, using a cutoff Coulombic efficiency value of 95%.^42,43,50^ The representative Coulombic efficiencies of each electrolyte are summarized in Table S1. The supercapacitor cell utilizing [(1-a-pyrr)­N_111_BH_2_]­TFSI achieves a maximum operating voltage window of 3.67 ± 0.06 V, comparable to the operating voltage of the [(1-m-pyrr)­N_111_BH_2_]­TFSI cell (3.55 ± 0.07 V). The wide operating voltages for these two BILs can be attributed to the strong coordination between the boron center and the nitrogen donors from the conjugated groups, as well as the inherent electrochemical stability of the pyrrolidinium ligand, in agreement with previous research on pyrrolidinium-based ILs.^51,52^ Notably, the ammonium- and pyrrolidinium-based BILs show significantly higher operating voltages than the organic electrolyte acetonitrile (ACN)/1 M tetrabutylammonium hexafluorophosphate ([TBA]­PF_6_) (2.90 ± 0.00 V) tested under the same symmetric cell configuration with CNFP electrodes.

After an operating voltage was determined, CV and GCD experiments were conducted to validate capacitive behavior and comparatively assess the electrochemical performance of each supercapacitor cell. Figure 4a shows the scan-rate dependent CV profiles of [(1-m-pyrr)­N_111_BH_2_]­TFSI in the potential range of 0–3.6 V at various rates from 5–200 mV s^–1^. Three successive scans were performed at each scan rate, and the CV profiles exhibited little change, indicating good infusion and wetting of the CNFP electrodes by BILs. The CV curves show a sharp increase of current at the beginning of their charge cycle due to the rapid formation of the electric double layer.^53^ The curves maintain their rectangular shape without any obvious reduction–oxidation peaks or distortions with escalating scan rates from 5 to 200 mV s^–1^, confirming good capacitive behavior. GCD experiments further confirm the typical capacitive behaviors of each BIL electrolyte. As shown in Figure 4b, the GCD profiles of [(1-m-pyrr)­N_111_BH_2_]­TFSI obtained with increasing current density from 0.1 A g^–1^ to 1.5 A g^–1^ manifest close to linear charge–discharge response as a function of time and symmetrical triangular profiles, indicating electrostatic double-layer charge-storage behavior. At all current densities, [(1-m-pyrr)­N_111_BH_2_]­TFSI exhibits longer charge–discharge duration times compared to the other BILs, indicating its higher specific capacity (Figure S7 and Table S2). Moreover, all BILs show longer charge–discharge duration times than ACN/1 M [TBA]­PF_6_, providing evidence that the charge-storage capacity using a BIL electrolyte improves compared to that of this commercial organic electrolyte.

**Figure 4:**
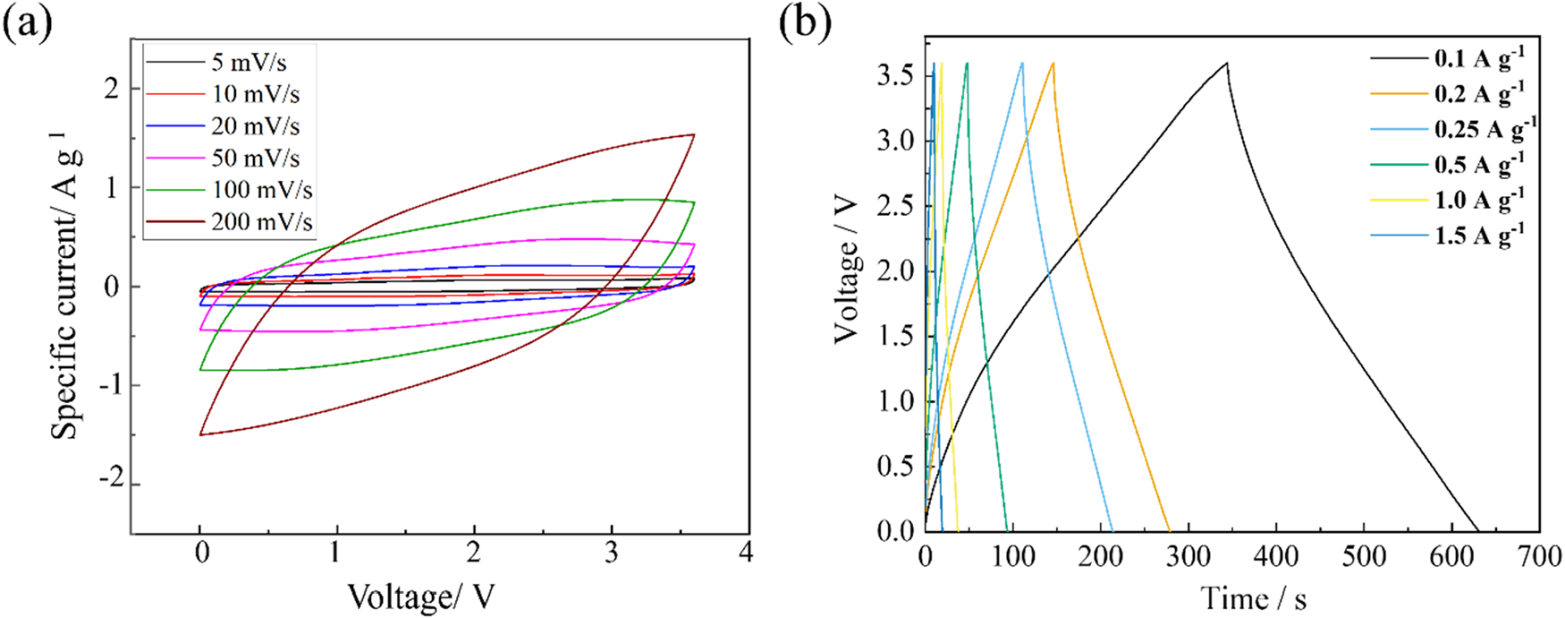
(a) Cyclic voltammetry (CV) curves of [(1-m-pyrr)­N_111_BH_2_]­TFSI up to a constant voltage of 3.6 V with varying scan rate ranging from 5 to 200 mV s^–1^. The 3rd-cycle CV profile was depicted at each scan rate. (b) Galvanostatic charge–discharge (GCD) curves of [(1-m-pyrr)­N_111_BH_2_]­TFSI with varying current densities ranging from 0.1 to 1.5 A g^–1^.

Based on the GCD measurements, the specific capacitance of each BIL was determined from 0.05 to 1.5 A g^–1^ current density. These data are presented in Figure 5. The specific capacitance of all BIL electrolytes decreases as the current density increases (Figure 5a), indicating insufficient time for the electrolyte ions to migrate to/from the electrode surface.^48^ The following trend is observed for the specific capacitance of the BIL electrolytes studied in this work across all current densities tested: [(1-m-pyrr)­N_111_BH_2_]­TFSI > [(1-a-pyrr)­N_111_BH_2_]­TFSI > [N_112_N_112_BH_2_]­TFSI > [(1-a-imid)­N_111_BH_2_]­TFSI. This trend suggests a trade-off between specific capacitance and current density due to each electrolyte’s diffusion kinetics. At higher current density, the specific capacitance of the four BILs show increasing disparity, exhibiting 7.9, 6.8, 6.4, and 5.3 F g^–1^ at 0.5 A g^–1^ for [(1-m-pyrr)­N_111_BH_2_]­TFSI, [(1-a-pyrr)­N_111_BH_2_]­TFSI, [N_112_N_112_BH_2_]­TFSI, and [(1-a-imid)­N_111_BH_2_]­TFSI, respectively. Notably, a cell assembled with [(1-m-pyrr)­N_111_BH_2_]­TFSI delivers a specific capacitance as high as 9.1 F g^–1^ at 0.02 A g^–1^. The higher specific capacitance for [(1-m-pyrr)­N_111_BH_2_]­TFSI should be mainly ascribed to its higher ionic conductivity, facilitating the formation of the electric double layer at the electrode surface as a function of the applied voltage. Although, the specific capacitance of all BILs surpasses that of ACN/1 M [TBA]­PF_6_ measured under the same conditions (Figure S8), the limited capacitance is likely associated with the ion accessibility and degree of the BILs cation-TFSI anion electrosorption at/within the mesoporous electrodes. Ongoing research is focused on elucidating the impact of the BIL and CNFP structures on the capacitive performance of assembled cells.

**Figure 5:**
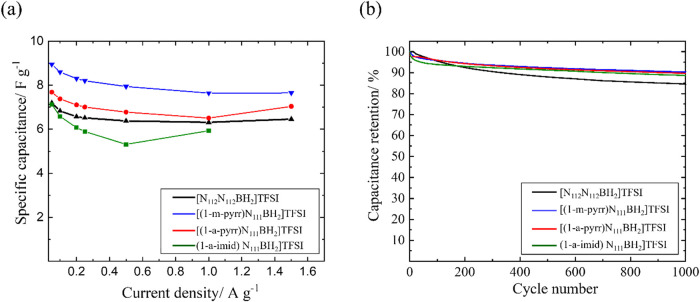
(a) Specific capacitance of each BIL electrolyte. The specific capacitance is calculated based on the total mass of two carbon nanofoam paper electrodes (CNFP). Data were collected across a range of current densities from 0.05 to 1.5 A g^–1^. (b) Comparison of long-term operational stabilities among each BIL electrolyte during 1000 galvanostatic charge–discharge (GCD) cycles at a constant current density of 0.5 A g^–1^.

It is interesting to note the emergence of pseudoredox peaks in the CV curves of [(1-a-pyrr)­N_111_BH_2_]­TFSI (see Figure S6b). Further analysis of the electrochemical data (Figure S9) suggests that these peaks result from a combination of surface-limited and diffusion-controlled double-layer charge-storage processes. Such peaks could be due to electrochemically generated species comprising polymerizable allyl-moieties and/or organic/inorganic species within the [(1-a-pyrr)­N_111_BH_2_]­TFSI, and can possibly be the reason why this BIL has the second highest specific capacitance.

Long cycle life is another important feature for practical supercapacitors. The durability of the BIL electrolytes was tested over 1000 GCD cycles at a current density of 0.5 A g^–1^; the corresponding evolution of the capacitance is shown in Figure 5b. These data show higher cycling durability of the pyrrolidinium- and imidazolium- substituted BILs compared to ammonium (N_112_). This enhanced performance can be attributed to the inherent stability of the ligand structure, offering more resilience during electrochemical cycling. After 1000 cycles, the GCD curves of all four BIL electrolytes maintain their triangular shapes (data not shown), giving good specific capacitance retention values of 90.3, 89.6, 88.6, and 84.5% for [(1-m-pyrr)­N_111_BH_2_]­TFSI, [(1-a-pyrr)­N_111_BH_2_]­TFSI, [(1-a-imid)­N_111_BH_2_]­TFSI, and [N_112_N_112_BH_2_]­TFSI, respectively. The life span of ionic-liquid-based supercapacitors is typically shorter than that of both aqueous and organic electrolyte-based supercapacitors due to the lower conductivity and higher viscosity of ILs. Furthermore, high-surface-area carbon materials, including the CNFP electrodes, are more susceptible to secondary reactions, which could lead to the pore blockage, hinder ion access to the surface, and negatively impact cell performance.^54^ However, it is important to highlight that [(1-a-pyrr)­N_111_BH_2_]­TFSI exhibits >90% capacitance retention at a notably high voltage (3.6 V), and still maintains 80% capacitance after 3000 charge–discharge cycles (see Figure S10) (note: a supercapacitor is considered degraded when its initial capacitance decreases by 20% from its initial value^55,56^ ).

To understand the nature of the electrode/electrolyte interface, preliminary X-ray photoelectron spectroscopy (XPS) experiments were performed on a Pt electrode after cycling it in a three-electrode configuration with a representative BIL ([(1-a-pyrr)­N_111_BH_2_]­TFSI). XPS was performed on the cycled Pt electrode dried under a vacuum. The C 1s, N 1s, F 1s, and S 2p binding energy regions (Figure S11) observed on the electrode outermost surface indicate assembly of the BIL cations and anions (note: the observed peaks in Figure S11 match those of the virgin BIL, data not shown). Of particular interest are the peaks in the F 1s and S 2p regions, which are only attributable to the TFSI anion. The single F 1s peak, and the S­(2p_1/2_/2p_3/2_) doublet with the 2p_3/2_ centered at 167.9 eV, both suggest single speciation of the TFSI anion in this outermost layer (adjacent to the bulk electrolyte) covering the Pt electrode.^57,58^ Next, soft-ion (C60) sputtering was performed through this surface layer to evaluate the layer between the bulk electrolyte and the Pt electrode. Approximately, 400 nm of this region was systematically sputtered through and analyzed at 20 nm intervals. Analysis of the C 1s spectra through this region reveals that the peak at 285.5 eV (attributed to the C=C of the allyl group on the boronium cation) gradually decreases while the peak at 284.5 eV (attributed to C–C species within the BIL) increases (Figure S12). These data suggest that the allyl-substituted boronium cation between the bulk electrolyte and the Pt surface likely polymerized as electrons are driven to the cathode. While this depth profile does not fully explain the nature of this layer (i.e., thickness, speciation, etc.), it does support two preliminary conclusions: (i) a polymerized BIL layer likely exists between the bulk electrolyte and the Pt surface, and (ii) this layer is ionically conductive and provides surface integration. Such a passivation layer could limit electrolyte access to microporous regions of CNFP electrodes, explaining observed reductions or limits of specific capacitance. Conversely, the presence of a stable and ionically conductive polymerized BIL layer at the electrode surface could enhance the cycling stability of BIL-based supercapacitors. These preliminary findings highlight the importance of studying the electrode–electrolyte interface and pave the way for future studies of this interface, which will be critical to future development of BIL-based supercapacitors.

Energy density and power density are two crucial metrics to determine the quality of supercapacitors. We calculated the energy and power density based on the specific capacitance obtained from GCD curves at each current density. These results are summarized as Ragone plots in Figure 6, with the corresponding calculated values provided in Table S3. The supercapacitor cells assembled with [(1-m-pyrr)­N_111_BH_2_]­TFSI or [(1-a-pyrr)­N_111_BH_2_]­TFSI demonstrate superior performance compared with the ammonium- and imidazolium-based BILs. [(1-m-pyrr)­N_111_BH_2_]­TFSI achieves a maximum energy density of 16.3 Wh kg^–1^ at a power density of 36.4 W kg^–1^, and maintains an energy value of 13.8 Wh kg^–1^ at a power density of 9.1 kW kg^–1^. Comparably, cells assembled with [(1-a-pyrr)­N_111_BH_2_]­TFSI achieves similar energy density ranging from 12.7–14.8 Wh kg^–1^ at higher power density of 13.9 kW kg^–1^ and 36.3 W kg^–1^, respectively. The higher power density presented by [(1-a-pyrr)­N_111_BH_2_]­TFSI is mainly credited to its faster discharge time compared to [(1-m-pyrr)­N_111_BH_2_]­TFSI. Overall, the power density presented by BIL electrolytes surpasses that of the aprotic organic electrolyte tested under the same cell configurations (Figure S13a and Table S4) and other IL electrolytes in carbon-based supercapacitors, while exhibiting the comparable energy density performance to those previously reported in the literature (Figure S13b). While the energy density of the BILs may not be superior compared to other ILs or certain organic electrolyte-based supercapacitors, the less pronounced drop in the Ragone plot suggests their ability to deliver steady energy at a high power density. This capability may be due to the formation of a protective surface passivation layer by a BIL electrolyte that still facilitates the ion and electron transfer to the electrode surface.

**Figure 6:**
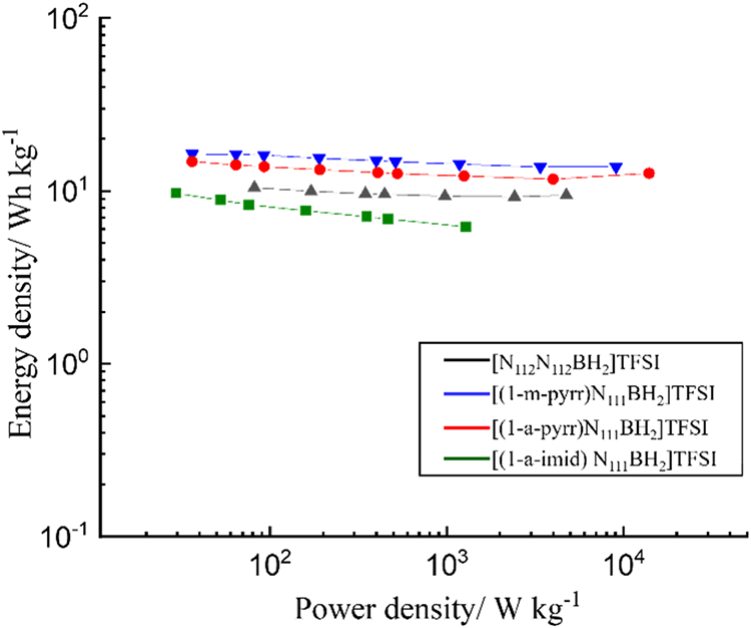
Ragone plot depicting energy and power density of boronium ionic liquids (BILs) electrolytes; [N_112_N_112_BH_2_]­TFSI, [(1-m-pyrr)­N_111_BH_2_]­TFSI, [(1-a-pyrr)­N_111_BH_2_]­TFSI, and [(1-a-imid)­N_111_BH_2_]­TFSI.

Table 1 summarizes the capacitance, maximum energy and power density, and capacitance retention of the four BIL electrolytes investigated in this study. Other literature on IL electrolytes in carbon-based supercapacitors is collated, as shown in Table S5, listing the specific capacitance, operating cell voltage, energy/power density, and specific capacitance retention after cycling. In general, BILs exhibit excellent power density and satisfactory energy density with an anticipated advantage as (quasi-) solid-state electrolytes benefiting from their polymerizable functional groups.

**Table 1:** Comparison of Supercapacitor Performance of Boronium Ionic Liquid (BILs) Electrolytes Tested in This Study

BILs	specific capacitance, F g^–1^ (at 0.5 A g^–1^)	power density (W kg^–1^)	energy density (Wh kg^–1^)	capacitance retention, % (after 1000 cycles)
[N_112_N_112_BH_2_]TFSI	6.4	4740.7	10.4	84.5
[(l-m-pyrr)N_111_BH_2_] TFSI	7.9	9067.5	16.1	90.3
[(l-a-pyrr)N_111_BH_2_] TFSI	6.8	13,930.5	13.8	89.6
[(l-a-imid)N_111_BH_2_] TFSI	5.3	1284.2	8.3	88.6

Finally, the electrochemical performance of BILs-CNFP cells was investigated at elevated temperature (40 and 50 °C). As seen in Figure 7a, the specific capacitance of an ammonium-based BIL, [N_112_N_112_BH_2_]­TFSI trends upward with increasing temperature from 25 to 50 °C, though the values are within the error of the measurement. This increased capacitance is attributed to the facilitated ion transport and charge carrier mobility, thereby accelerating the formation of electric double layers. However, as temperature increases, the operating voltage window of [N_112_N_112_BH_2_]­TFSI simultaneously decreases ∼10% (from 3.25 to 2.9 V), leading to the same or slightly lower energy and power density (see Figure 7b and Table S6). The capacitive performance of the [(1-a-pyrr)­N_111_BH_2_]­TFSI was similarly evaluated at 40 and 50 °C, generally revealing the same performance trends (see Figure S14). Ultimately, the temperature-control studies show that while BILs do remain stable as supercapacitor electrolytes at elevated temperatures (up to 50 °C), there is no significant performance advantage obtained.

**Figure 7:**
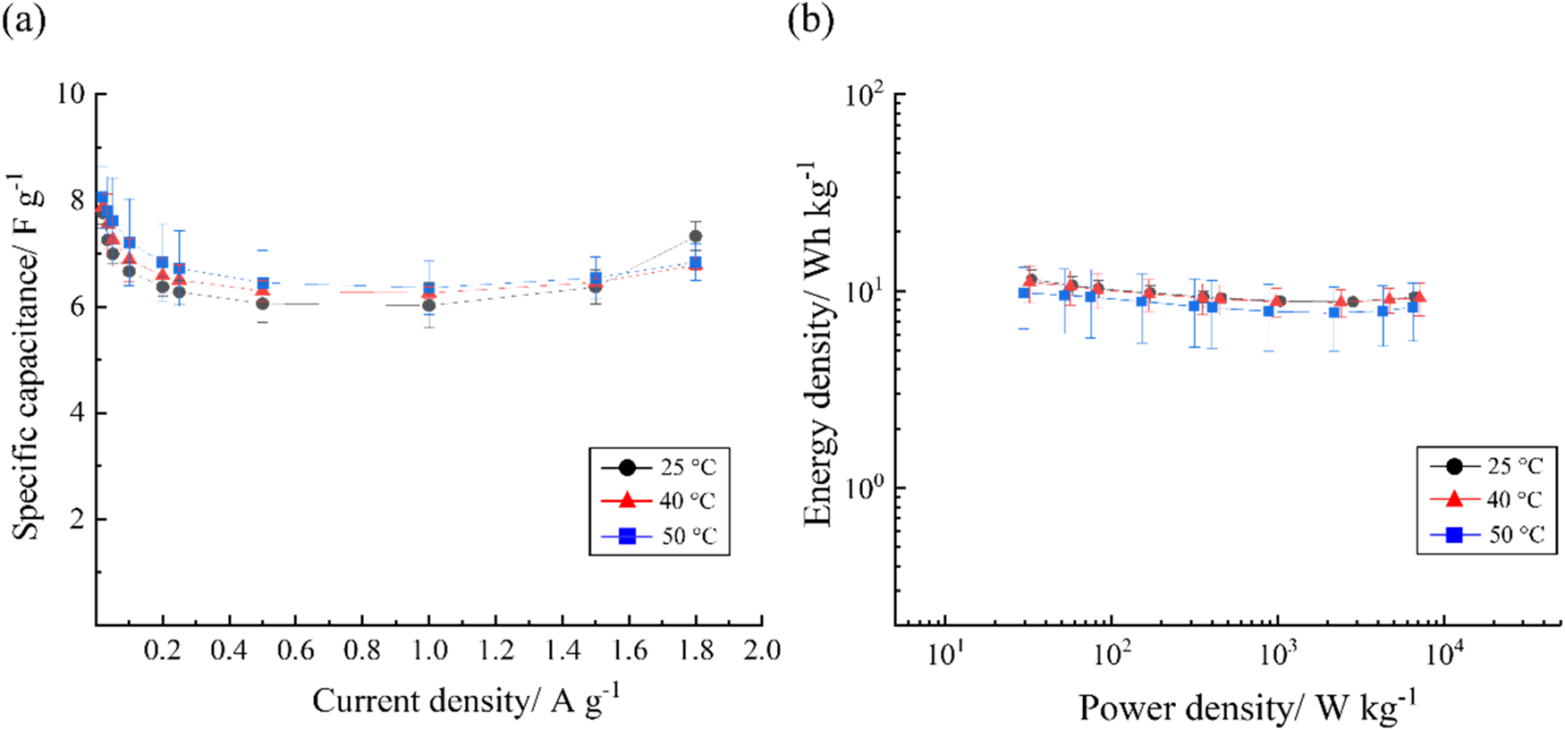
(a) Specific capacitance and (b) Ragone plot depicting energy and power density of [N_112_N_112_BH_2_]­TFSI at different temperatures from 25 to 50 °C.

The fundamental studies presented in this report offer exciting benchmark performance metrics for a suite of BIL electrolytes upon which “best in class” performance BILs could be designed in the future. While the BILs in this study are prepared on a small (50–150 g) scale, they are targeted for variety in ligand structure and without serious consideration for minimizing cost. However, because the synthesis is straightforward, scalable, and delivers near quantitative yield, it is reasonable to expect that once a BIL structure is optimized to maximize electrochemical performance, a successful path toward a cost-effective, commercialized BIL supercapacitor system can be achieved.

## Conclusions

This study systematically evaluates for the first time a series of BILs as electrolytes in symmetric electrochemical double-layer supercapacitors coupled to carbon nanofoam paper (CNFP) electrodes. We elucidate the key capacitive characteristics of four BIL-TFSI electrolytes, capitalizing on their wide operating voltage windows and high electrochemical stabilities, thereby opening new avenues for the functionalization of BILs as effective supercapacitor electrolytes. Complementary electrochemical techniques (CV, EIS, GCD) indicate good capacitive behavior, supercapacitive performance, and satisfactory long-term durability of each BIL. Of the electrolytes tested, the pyrrolidinium-substituted BILs offer the highest energy and power density of supercapacitor cells, benefiting from the extended operating voltage to ∼3.7 V. Overall, these BILs exhibit excellent power density and sufficient energy density with the advantage in delivering the energy steadily at high power density. Moreover, polymerizable [(1-a-pyrr)­N_111_BH_2_] derivatized BILs may offer the potential of adopting a solid or pseudosolid state and implementation in flexible wearable supercapacitors. Finally, the specific capacitance, energy density, and power density of ammonium- and pyrrolidinium-based BILs exhibit a delicate dependence on temperature intended to facilitate the diffusion kinetics of BILs, confirming thermal resilience but no significant performance advantage. Future work is directed toward investigating electrode architectures and electrode/electrolyte interfaces to achieve higher energy and power density.

## Supplementary Material



## Notes

Additional data including synthesis and characterization of [(1-a-imid)­N_111_BH_2_]­TFSI, XPS experimental information, tabulated electrochemical performance of BILs and other ILs-based supercapacitors in the previous works, cyclic voltammetric (CV) curves, galvanostatic charge–discharge curves (GCD), and specific capacitance of BILs, XPS spectra of [(1-a-pyrr)­N_111_BH_2_]­TFSI, Ragone plot depicting BILs and other carbon-based supercapacitors, temperature effect on the electrochemical performance of [(1-a-pyrr)­N_111_BH_2_]­TFSI. (PDF)
