# Supplementary material for: Boronium Ionic Liquids for High-Voltage Supercapacitors
Source: ACS Appl Eng Mater. 2025 Nov 24;3(12):4560–8. doi: 10.1021/acsaenm.5c00888 (PMC12750520; doi:10.1021/acsaenm.5c00888)
Supplement: Supplementary file 1 [file em5c00888_si_001.pdf]

Supporting Information

Boronium Ionic Liquids for High-Voltage Supercapacitors

Whirang Cho [a], Christopher D. Stachurski [a], Zachary G. Neale[b], Miaomiao Ma[c], Margaret E. Crowley[d], Matthias Zeller[e], James H. Davis Jr.[d], Paul C. Trulove [a], David P. Durkin [a,*]

[a] Department of Chemistry, U. S. Naval Academy, Annapolis, Maryland 21402, USA
[b] Surface Chemistry Branch, U.S. Naval Research Laboratory, Washington, DC 20375, USA
[c] Carderock Division, Naval Surface Warfare Center, Bethesda, MD 20817, USA
[d] Department of Chemistry, University of South Alabama, Mobile, Alabama 36688, USA
[e] Department of Chemistry, Purdue University, West Lafayette, Indiana 47907, USA

[*] Corresponding author: Dr. David P. Durkin, durkin@usna.edu

Materials. Toluene (Fisher Scientific, ACS Grade), diethyl ether (Pharmco, reagent grade ACS), chloroform (Pharmco, reagent grade ACS), ethyl acetate (Sigma Aldrich, 99.8%), acetonitrile (Pharmco, reagent grade ACS), borane trimethylamine complex (Sigma Aldrich, 97%), iodine (Sigma Aldrich, > 99.8%), and 2,6-di-tert-butyl-4-methylphenol (BHT, Sigma Aldrich, > 99%,) were used as received. 1-allylimidazole (Sigma Aldrich, > 99%) was freshly distilled prior to use. Lithium bis(trifluoromethanesulfonyl) imide (Li[TFSI]), magnesium sulfate ($MgSO_4$, Alfa, reagent grade) and silver triflate (Sigma Aldrich, > 99%) were used as received. Deuterated acetonitrile (acetonitrile-d3, Fisher) was used for NMR analysis.

Synthesis of [(1-a-imid)$N_{111}BH_2$]TFSI

Following a modified procedure[1-3], [(1-a-imid)$N_{111}BH_2$]TFSI ("Durkanium") was synthesized. To a two-neck round bottom flask, 18 g (0.25 mol) of borane trimethylamine complex was dissolved in 150 ml of toluene and stirred in an ice bath. 29.77 g (0.12 mol) of crystalline I_2 was then slowly added into the chilled solution, which developed a dark red/brown color immediately. To this, 0.1 wt% 2,6-di-tert-butyl-4-methylphenol (a stabilizing agent to prevent autopolymerization) and 26.68 g (0.25 mol) of distilled 1-allylimidazole were added and stirred overnight. The product (white precipitate) was collected by filtration and washed with diethyl ether. The solids were then allowed to dry under vacuum overnight. Anion exchange was performed by dissolving the iodide salt in an aqueous solution of 1.5-fold excess Li[TFSI]. After stirring overnight, the final product, [(1-a-imid)$N_{111}BH_2$]TFSI was collected, dried over $MgSO_4$, and filtered. The clear, colorless ionic liquid solution was then transferred to 150 ml round bottom flask, dried by rotary evaporation, and then further dried in vacuo at room temperature overnight. Following synthesis and purification, [(1-a-imid)$N_{111}BH_2$]TFSI structure was confirmed using NMR and single-crystal X-ray

diffraction. The thermal stability, glass transition temperature (*Tg*), and electrochemical performance were then investigated.

Crystal Structure Determination

The crystal structure of [(1-a-imid)$N_{111}BH_2$]I was determined by single-crystal X-ray diffraction using a Bruker Quest diffractometer with a fixed χ angle, a Mo Kα wavelength (λ = 0.71073 Å) sealed tube fine focus X-ray tube, single-crystal curved graphite incident beam monochromator, a Photon II area detector, and an Oxford Cryosystems low-temperature device. Examination and data collection were performed at 150 K. Data were collected, reflections were indexed and processed, and the files scaled and corrected for absorption using APEX5 (Apex, SAINT, Bruker AXS Inc, Madison, WI, USA)] and SADABS[4]. The space group was assigned using XPREP within the SHELXTL suite of programs[5] and solved by dual methods using ShelXT[6] and refined by full matrix least squares against F^2 with all reflections using Shelxl2019[7] using the graphical interface Shelxle[8]. H atoms attached to carbon were positioned geometrically and constrained to ride on their parent atoms. C-H bond distances were constrained to 0.95 Å (for aromatic C-H groups). U_{iso}(H) values were set to 1.2 times multiple of U_{eq}(C). The cation is disordered by a pseudo-two-fold rotation of the five-membered ring. The disorder extends to the entire cation. For the trimethyl amine group, the major moiety was refined as additionally split. Equivalent disordered moieties were restrained to have similar geometries. U_{ij} components of ADPs for disordered atoms closer to each other than 2.0 Å were restrained to be similar. Subject to these conditions the occupancy ratio refined to 0.9272(12) to 0.0727(12) for the two-fold disorder. The trimethyl amine disorder refined to 0.746(3) to 0.181(3) to 0.0727(12).

X-ray photoelectron spectroscopy (XPS)

The XPS spectra were collected using a Physical Electronics PHI-5000 VersaProbe™ II Scanning XPS Microprobe (monochromatic Al Kα X-ray source, 1486 eV) operated at 15 kV and 50 W under ultrahigh vacuum conditions. Low-resolution spectra were collected to survey the elements present in the surface region followed by high-resolution multiplexes of the identified elements. All spectra were collected at an electron emission angle of 45°. Sputtering was conducted using 20 keV C60 with a raster area of 3 mm × 3 mm in intervals of 2 min up to 40 min. The sputtering rate was calibrated using a 1000 Å thick SiO_2 film on silicon substrate and was estimated to be around 10.5 nm of SiO_2 per minute. Final adjustment to the binding-energy scale was made by calibrating the C1s line for C-C to 284.5 eV. Deconvolution of multiple peaks for each region was performed using the manufacturer provided Multipak software version 9.9.0.8 employing a combined Gaussian-Lorentzian line shape and an iterated Shirley model for the background. XPS samples were prepared by using a three-electrode setup consisting of a Pt wire working electrode, a platinum-mesh counter electrode, and a home-built Ag/Ag^+ reference electrode. To assemble the reference electrode, a 100 mM solution of silver triflate was prepared in [1-ethyl-3-methylimidazolium]TFSI and sealed in a glass capillary tube using a Vycor glass frit and silver wire contact. CV scans were performed between –3.5 V and 2.5 V.

Structure Table

	**[(1-a-imid)$N_{111}BH_2$]I.cif**
CCDC number	2462235
Empirical formula	$C_{33}H_{39}B_2N_3$
Moiety formula	$C_{24}H_{20}B \cdot C_9H_{19}BN_3$
Formula weight	499.29
Temperature [K]	150(2)
Crystal system	monoclinic
Space group (number)	$P2_1/n$
a [Å]	11.2903(4)
b [Å]	16.6498(5)
c [Å]	15.7371(5)
α [°]	90
β [°]	99.535(1)
γ [°]	90
Volume [Å^3]	2917.41(16)
Z	4
ρ_{calc} [gcm^{-3}]	1.137
μ [mm^{-1}]	0.065
F(000)	1072
Crystal size [mm^3]	0.450 × 0.420 × 0.350
Crystal colour	colorless
Crystal shape	Block
Radiation	MoK_α (λ=0.71073 Å)
2Θ range [°]	4.40 to 66.39 (0.65 Å)
Index ranges	$-17 \leq h \leq 17$<br>$-25 \leq k \leq 25$<br>$-24 \leq l \leq 24$
Reflections collected	79392
Independent reflections	79392<br>R_{int} = 0.0442<br>R_{sigma} = 0.0442

Completeness	99.7 %
Data / Restraints / Parameters	11147 / 666 / 512
Goodness-of-fit on F^2	1.022
Final R indexes [$I \geq 2\sigma(I)$]	R_1 = 0.0512<br>wR_2 = 0.1351
Final R indexes [all data]	R_1 = 0.0724<br>wR_2 = 0.1515
Largest peak/hole [eÅ^{-3}]	0.32/−0.29

Table S1. Summary of representative coulombic efficiency of acetonitrile (ACN)/1 M tetrabutylammonium hexafluorophosphate ([TBA]PF_6) and BILs electrolytes, [$N_{112}N_{112}BH_2$][TFSI], [(1-m-pyrr)$N_{111}BH_2$][TFSI], [(1-a-pyrr)$N_{111}BH_2$][TFSI] and [(1-a-imid) $N_{111}BH_2$][TFSI]. The highlighted yellow cells indicate a threshold voltage established through coulombic efficiency measurement (The final row represents the average and the standard deviation for each data point, with n = 3).

Limiting Voltage / V	$N_{112}N_{112}BH_2$	[(m-pyrr)$N_{111}BH_2$]	[(a-pyrr)$N_{111}BH_2$]	[(1-a-imid)$N_{111}BH_2$]	ACN/[TBA]PF_6
2.0	97.7	98.8	97.3	93.2	95.1
2.2	97.7	98.9	97.5	96.2	95.5
2.4	97.3	98.8	97.8	96.6	95.6
2.6	97.2	98.3	97.6	96.5	95.6
2.8	96.8	97.8	97.3	95.9	95.3
3.0	96.0	97.3	97.0	93.1	94.8
3.2	95.2	97.0	96.8	90.1	93.5
3.4	93.9	95.6	96.2	83.2	92.4
3.6	91.5	94.8	95.5	70.1	91.2
3.8	87.5	93.8	94.7	51.9	90.4
4.0	80.9	93.0	93.5	20.1	88.1
Average threshold V	3.30 ± 0.09 V	3.55 ± 0.07 V	3.67 ± 0.06 V	2.97 ± 0.11 V	2.94 ± 0.12 V

Table S2. Summary of charge-discharge duration time of galvanostatic charge-discharge curves (GCD) at the current density of 0.1 A g^{-1}

electrolytes	charge-discharge duration time (at 0.1 A g^{-1})
$[N_{112}N_{112}BH_2]$TFSI	440 s
$[(1\text{-m-pyrr})N_{111}BH_2]$ TFSI	630 s
$[(1\text{-a-pyrr})N_{111}BH_2]$ TFSI	540 s
$[(1\text{-a-imid})N_{111}BH_2]$ TFSI	370 s
(ACN)/1 M $[TBA]PF_6$	320 s

Table S3. Comparison of power and energy density of boronium ionic liquid (BIL) electrolytes at different current densities ranging from 0.05 to 1.5 A g^{-1}

Current density (A g^{-1})	$[N_{112}N_{112}BH_2]$TFSI		$[(1\text{-m-pyrr})N_{111}BH_2]$ TFSI		$[(1\text{-a-pyrr})N_{111}BH_2]$ TFSI		$[(1\text{-a-imid})N_{111}BH_2]$ TFSI	
	Power density (W kg^{-1})	Energy density (Wh kg^{-1})	Power density (W kg^{-1})	Energy density (Wh kg^{-1})	Power density (W kg^{-1})	Energy density (Wh kg^{-1})	Power density (W kg^{-1})	Energy density (Wh kg^{-1})
0.05	81.74	10.44	92.49	16.10	92.75	13.83	76.11	8.33
0.1	170.76	9.96	189.81	15.47	191.41	13.27	159.78	7.68
0.2	346.45	9.62	339.52	14.94	407.32	12.79	353.16	7.10
0.25	444.79	9.58	512.54	14.76	525.63	12.61	465.79	6.88
0.5	975.98	9.34	1180.48	14.29	1252.73	12.20	1284.20	6.20
1.0	2417.40	9.24	3384.61	13.76	3988.93	11.70	-	-
1.5	4740.74	9.46	9067.52	13.77	13930.49	12.67	-	-

Table S4. The power and energy density of ACN/[TBA]PF_6 at different current densities ranging from 0.05 to 1.5 A g^{-1}

**Current density (A g^{-1})**	**ACN/1 M [TBA]PF_6**	
	Power density (W kg^{-1})	Energy density (Wh kg^{-1})
0.05	72.93	6.78
0.1	146.89	6.53
0.2	297.72	6.43
0.25	374.49	6.41
0.5	773.75	6.42
1.0	1654.78	6.58
1.5	2664.70	6.83

Table S5. Capacitive performance of various ionic liquid-based electrolytes in supercapacitors reported in the previous works

Electrode	IL	Specific capacitance/F g $^{-1}$	Cell voltage/ V $^{-1}$	Energy density/ Wh kg $^{-1}$	Power density/ kW kg $^{-1}$	Cyclability/ retention	Ref
Porous carbon	EMI-BF_4	147	3	11.4	98	90 % (10,000)	9
Porous carbon	EMI-BF_4	147	4.0	20	3.1	97 % (1,000)	10
Carbon	EMI-TFSI	160	3.0	20	42		11
Porous carbon nanofiber	EMI-TFSI	180	3.5	80	0.4		12
Carbonized cellulose/ Activated carbon	BMPY-TFSI	84	3.0	21	41.6	92 % (10, 000)	13
Mesoporous carbon	Imidazolium-based IL	131	2.5	38	3.58	80 % (2,000)	14
Activated carbon	BMI-Cl (gel-polymer)	136	1.5	10.6	3.4	90 % (3, 000)	15
Porous carbon	EMI-BF_4 (gel-polymer)	48.4	3.5	94.1	0.35		16
Honeycomb-like carbon	EMI-BF_4	174		74	0.87	87 % (10,000)	17
Graphitized carbon nanosheets	EMIM-TFSI/EMIM-BF4 (80:20)	132		56	93	97.6 % (10,000)	18
rGO	SET_3-TFSI-GO	125.3		17.7	0.87	77 % (3000)	19
Activated carbon	PYR13-FSI (gel-polymer)	21	2.5	16	1.1	100 % (2,500)	20
Activated carbon	PYR14-FSI (gel-polymer)	150	3.5	36	1.17		21
Activated carbon	BMI-BF_4 (gel-polymer)	138	2.5	36	24.5	80 % (10, 000)	22

Table S6. Average values of power and energy densities of $[N_{112}N_{112}BH_2]TFSI$ electrolyte at different current densities ranging from 0.02 to 1.8 A g^{-1} (Specific capacitance, energy density, and power density represent the average and the standard deviation of each measured data point, n = 3)

**Current density ($A\ g^{-1}$)**	**Specific capacitance ($F\ g^{-1}$)**	**25 °C**		**40 °C**		**50 °C**	
		Power density ($W\ kg^{-1}$)	Energy density ($Wh\ kg^{-1}$)	Power density ($W\ kg^{-1}$)	Energy density ($Wh\ kg^{-1}$)	Power density ($W\ kg^{-1}$)	Energy density ($Wh\ kg^{-1}$)
0.02	7.77 ± 0.22	33.05 ± 1.55	11.43 ± 1.37	32.11 ± 2.36	11.08 ± 2.39	29.61 ± 4.64	9.80 ± 3.36
0.35	7.26 ± 0.18	58.14 ± 2.80	10.67 ± 1.16	56.48 ± 4.08	10.59 ± 2.10	52.08 ± 8.21	9.51 ± 3.43
0.05	7.00 ± 0.18	83.55 ± 4.12	10.28 ± 1.05	82.05 ± 4.20	10.18 ± 2.00	74.82 ± 11.87	9.32 ± 3.53
0.1	6.67 ± 0.16	170.90 ± 9.03	9.79 ± 0.88	167.71 ± 7.44	9.65 ± 1.80	152.21 ± 24.08	8.84 ± 3.40
0.2	6.38 ± 0.18	358.05 ± 21.69	9.35 ± 0.64	351.74 ± 9.20	9.22 ± 1.65	315.80 ± 50.58	8.36 ± 3.17
0.25	6.28 ± 0.22	458.06 ± 29.63	9.20 ± 0.55	449.37 ± 10.10	9.11 ± 1.59	401.56 ± 64.29	8.23 ± 3.11
0.5	6.06 ± 0.35	1040.85 ± 89.66	8.87 ± 0.32	981.22 ± 29.37	8.82 ± 1.50	881.86 ± 143.02	7.88 ± 2.91
1.0	6.02 ± 0.42	2833.83 ± 417.16	8.81 ± 0.25	2404.02 ± 104.45	8.76 ± 1.40	2180.02 ± 367.53	7.74 ± 2.75
1.5	6.37 ± 0.32	6656.73 ± 1830. 39	9.33 ± 0.39	4692.99 ± 316.13	9.02 ± 1.32	4299.89 ± 825.33	7.93 ± 2.68
1.8	7.33 ± 0.27			7132.95 ± 87.56	9.23 ± 1.75	6512.57 ± 1655.27	8.27 ± 2.70

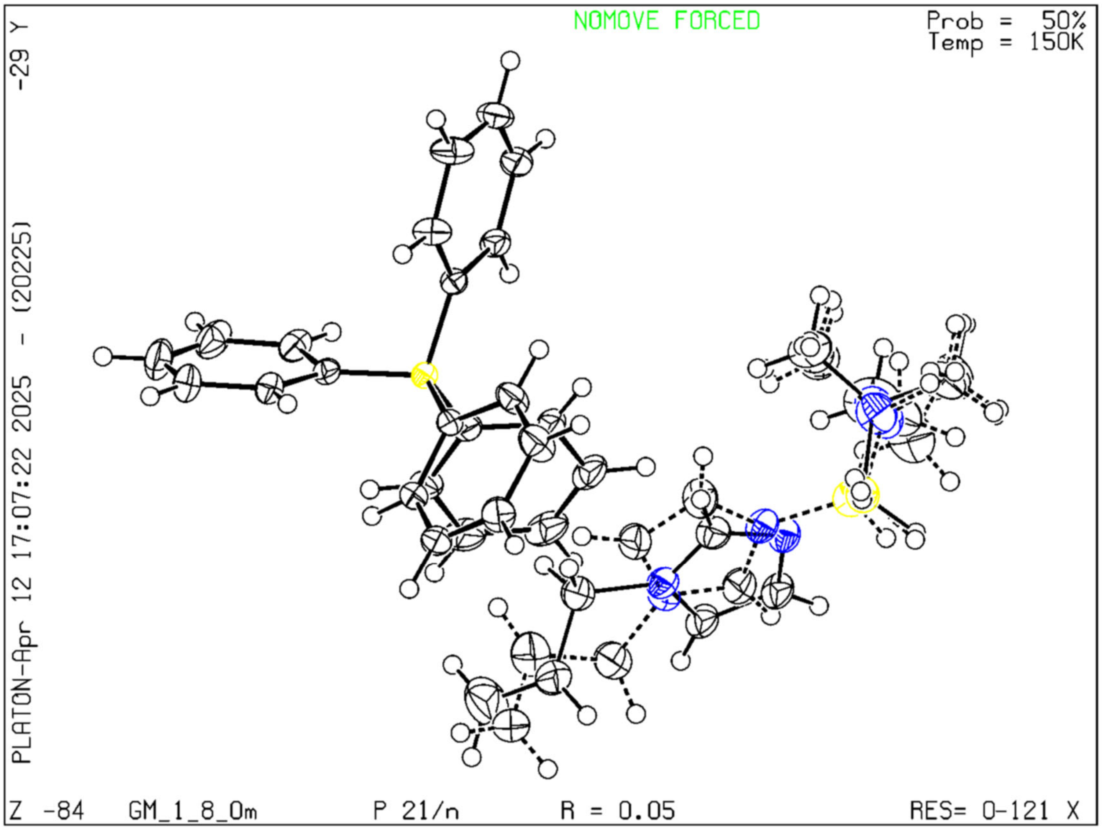

Figure S1. Molecular diagram of [(1-a-imid)$N_{111}BH_2$]I with non-hydrogen atoms represented by ellipsoids and hydrogen atoms as spheres of arbitrary size.

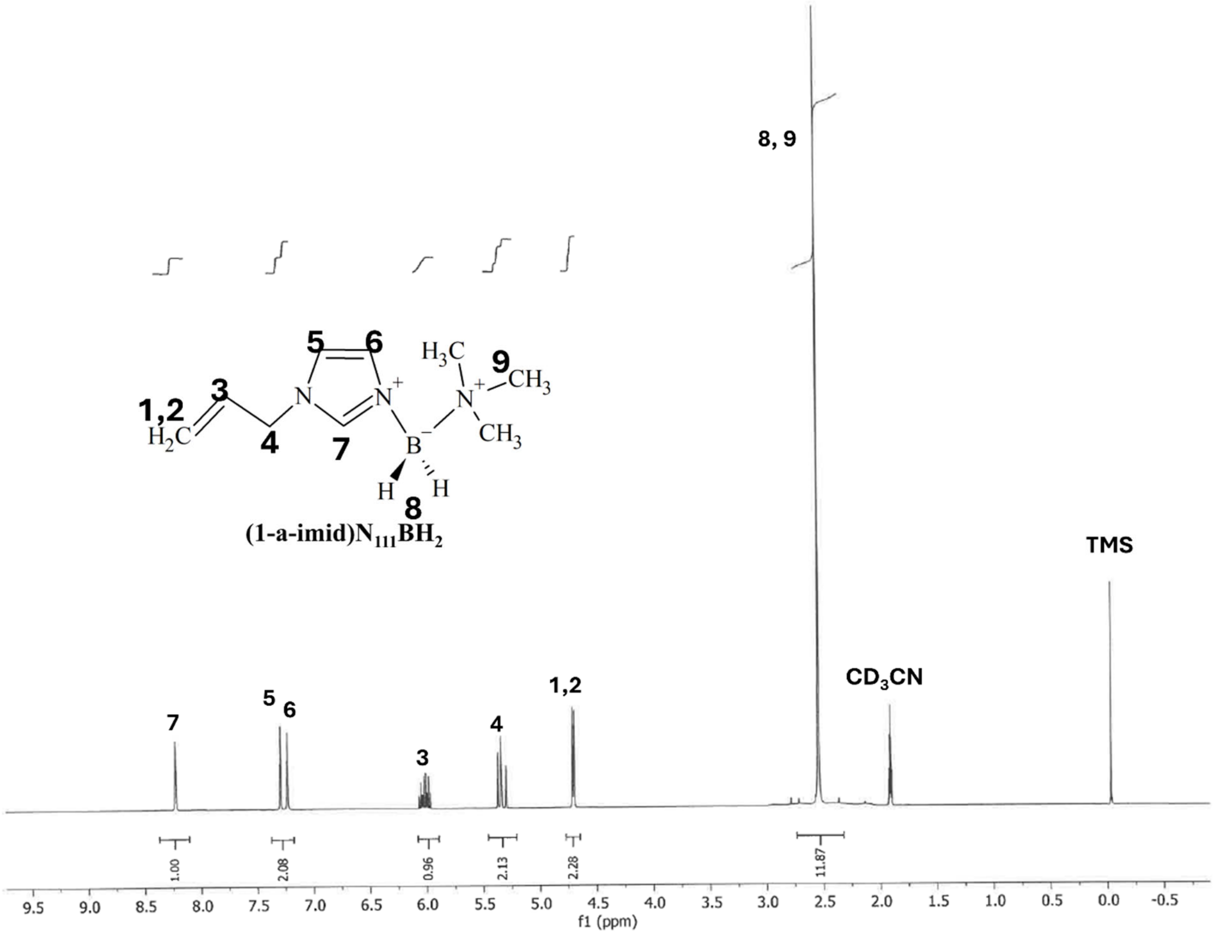

Figure S2. ^{1}H-NMR for [(1-a-imid)$N_{111}BH_2$]TFSI.

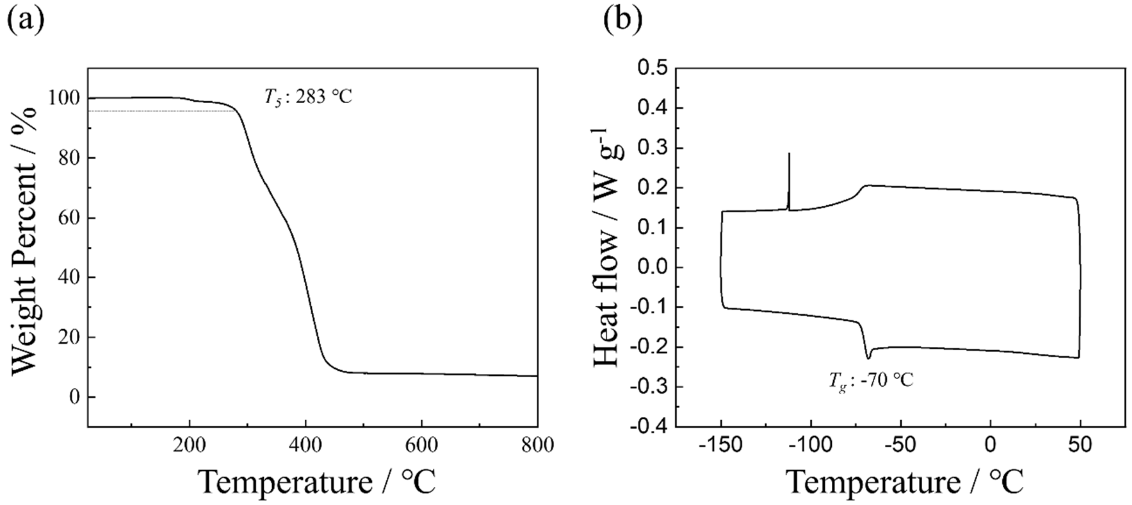

Figure S3. (a) Thermogravimetric analysis (TGA) and (b) differential scanning calorimetry (DSC) of [(1-a-imid)$N_{111}BH_2$]TFSI. Preconditioning for DSC was performed at 25 °C, before cooling to –150 °C then heating to 50 °C at a rate of 10 °C min^{-1}.

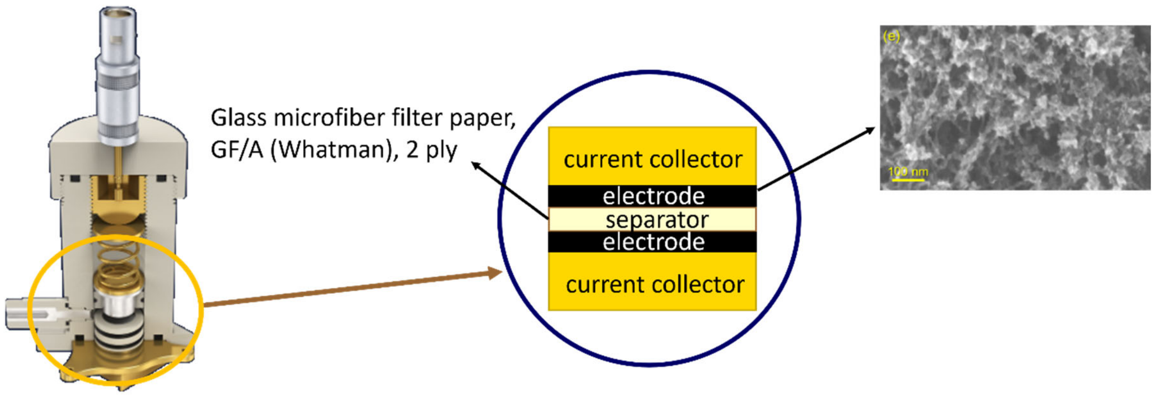

Figure S4. Schematic of two-electrode assembly using TSC battery cell (RHD instruments)

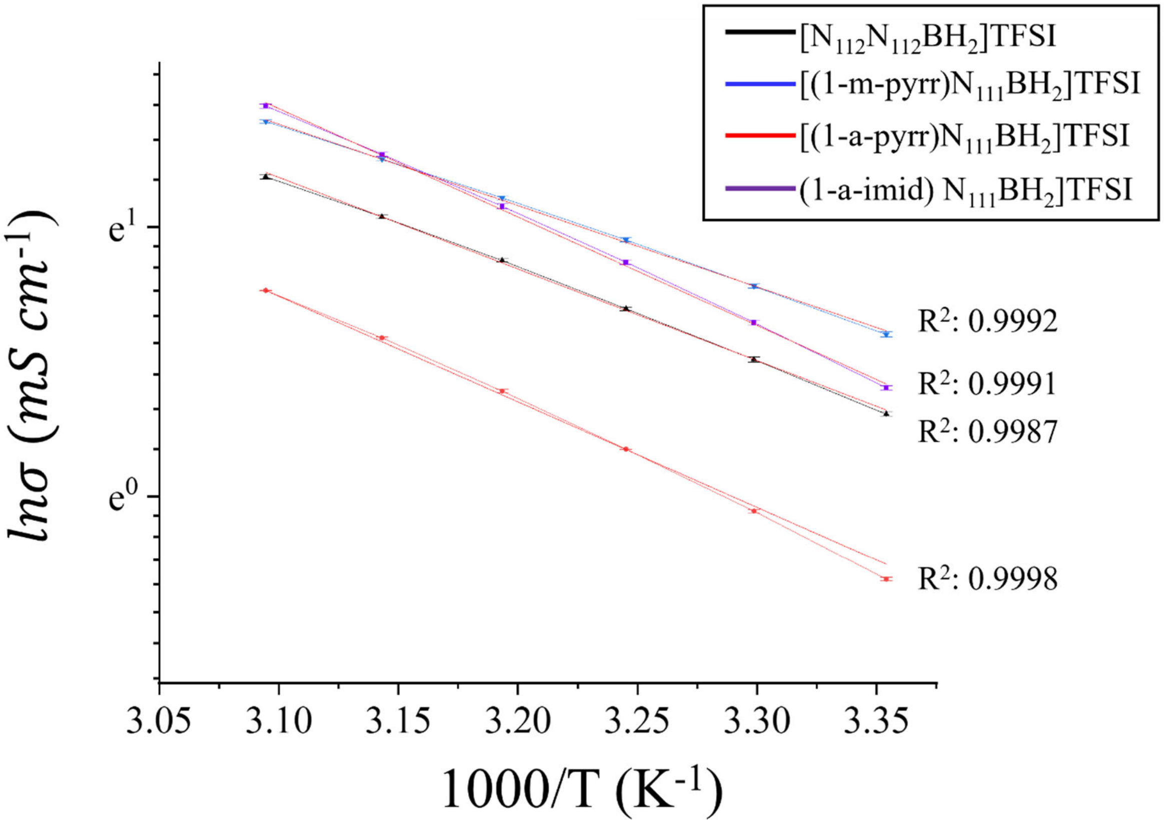

Figure S5. Temperature-dependent conductivity and Arrhenius fits (σ) of $[N_{112}N_{112}BH_2]TFSI$, $[(1\text{-a-pyrr})N_{111}BH_2]TFSI$, $[(1\text{-m-pyrr})N_{111}BH_2]TFSI$, and $[(1\text{-a-imid})N_{111}BH_2]TFSI$. Conductivity represents the average and the standard deviation of each measured data point obtained using different electrode assemblies run in parallel.

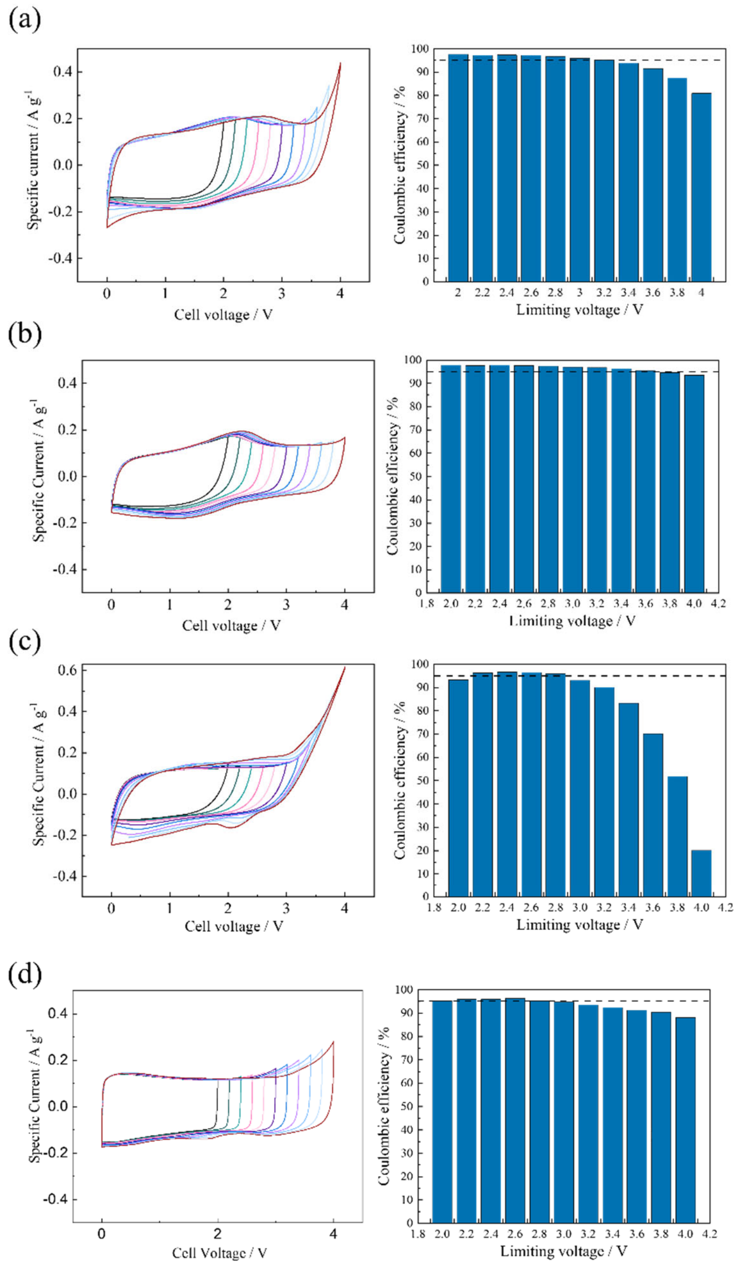

Figure S6. Cyclic voltammetric (CV) curves and the corresponding coulombic efficiency of (a) $[N_{112}N_{112}BH_2]$TFSI, (b) [(1-a-pyrr)$N_{111}BH_2$]TFSI, (c) [(1-a-imid)$N_{111}BH_2$]TFSI, and (d) ACN/[TBA]PF_6 at different voltages ranging from 2 to 4 V. Coulombic efficiency was calculated from the ratio between the amount of charge and discharge at each voltage.

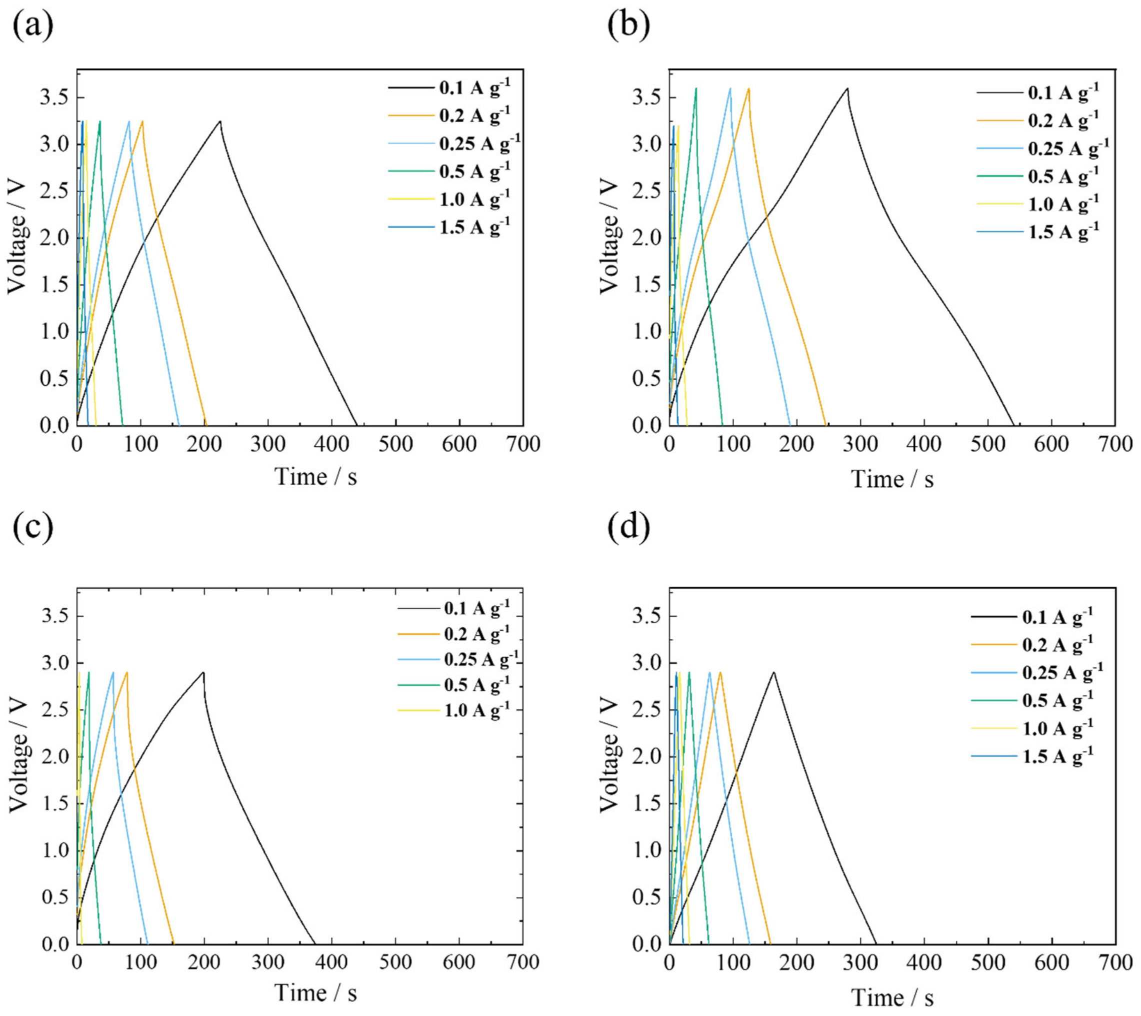

Figure S7. Galvanostatic charge-discharge curves (GCD) of (a) $[N_{112}N_{112}BH_2]$TFSI, (b) [(1-a-pyrr)$N_{111}BH_2$]TFSI, (c) [(1-a-imid)$N_{111}BH_2$]TSFI, and (d) ACN/[TBA]PF_6 at different current density ranging from 0.1 to 1.5 A g^{-1}.

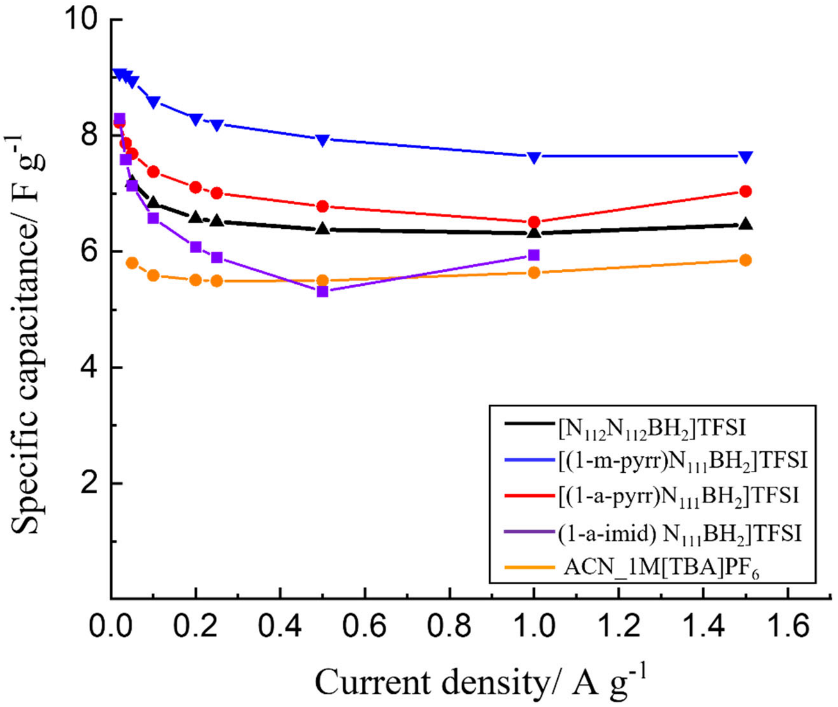

Figure S8. Comparison of specific capacitance at an applied current density ranging from 0.02 to 1.5 A g^{-1} of boronium ionic liquids (BILs) electrolytes; $[N_{112}N_{112}BH_2]$TFSI, [(1-m-pyrr)$N_{111}BH_2$]TFSI, [(1-a-pyrr)$N_{111}BH_2$]TFSI, and [(1-a-imid)$N_{111}BH_2$]TFSI and commercial organic electrolyte, ACN/1 M $[TBA]PF_6$.

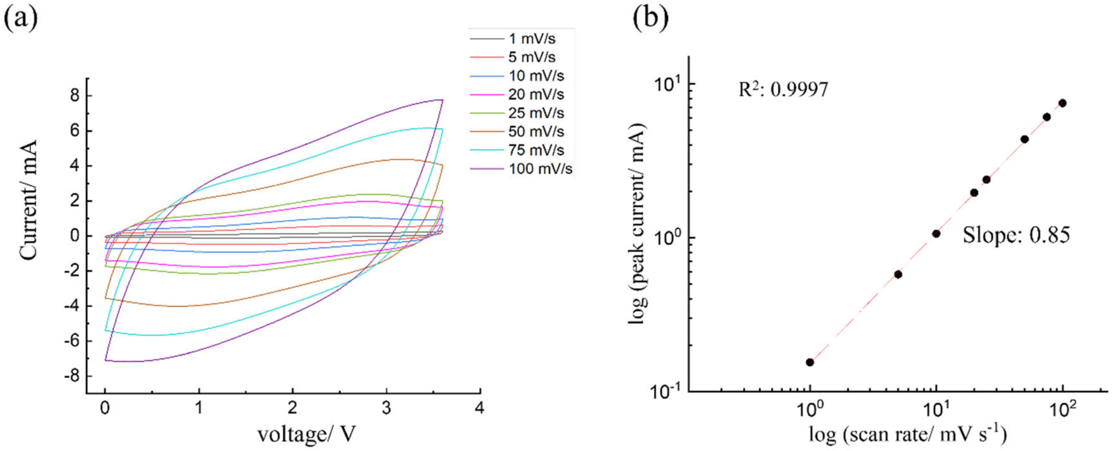

The plot of log (peak currents) of [(1-a-pyrr)$N_{111}BH_2$]TFSI at operating voltage 3.6 V against log (scan rate) reveals the linear increase to the power of 0.85. By applying the power-law relationship of currents to scan rate ($i = a\,\nu^b$), the slope of a log i vs. log ν plot manifests the rate-limiting step: diffusion controlled (b = 0.5) or surface-limited process (b = 1.0).[23]

Figure S9. The measured current (i) of [(1-a-pyrr)$N_{111}BH_2$]TFSI at a selected potential (V) follows a power-law relation with the scan rate, ν. $i = a\,\nu^b$ where b is the slope of a plot of log i vs. log ν.

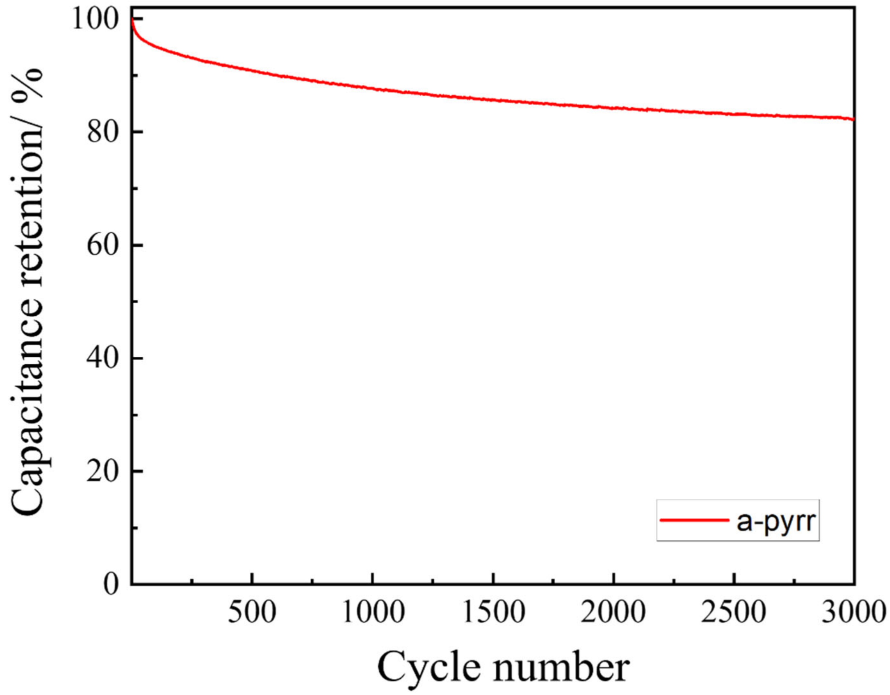

Figure S10. Long-term operational stability of [(1-a-pyrr)$N_{111}BH_2$]TFSI during 3000 galvanostatic charge-discharge (GCD) cycles at a constant current density of 0.5 A g^{-1}.

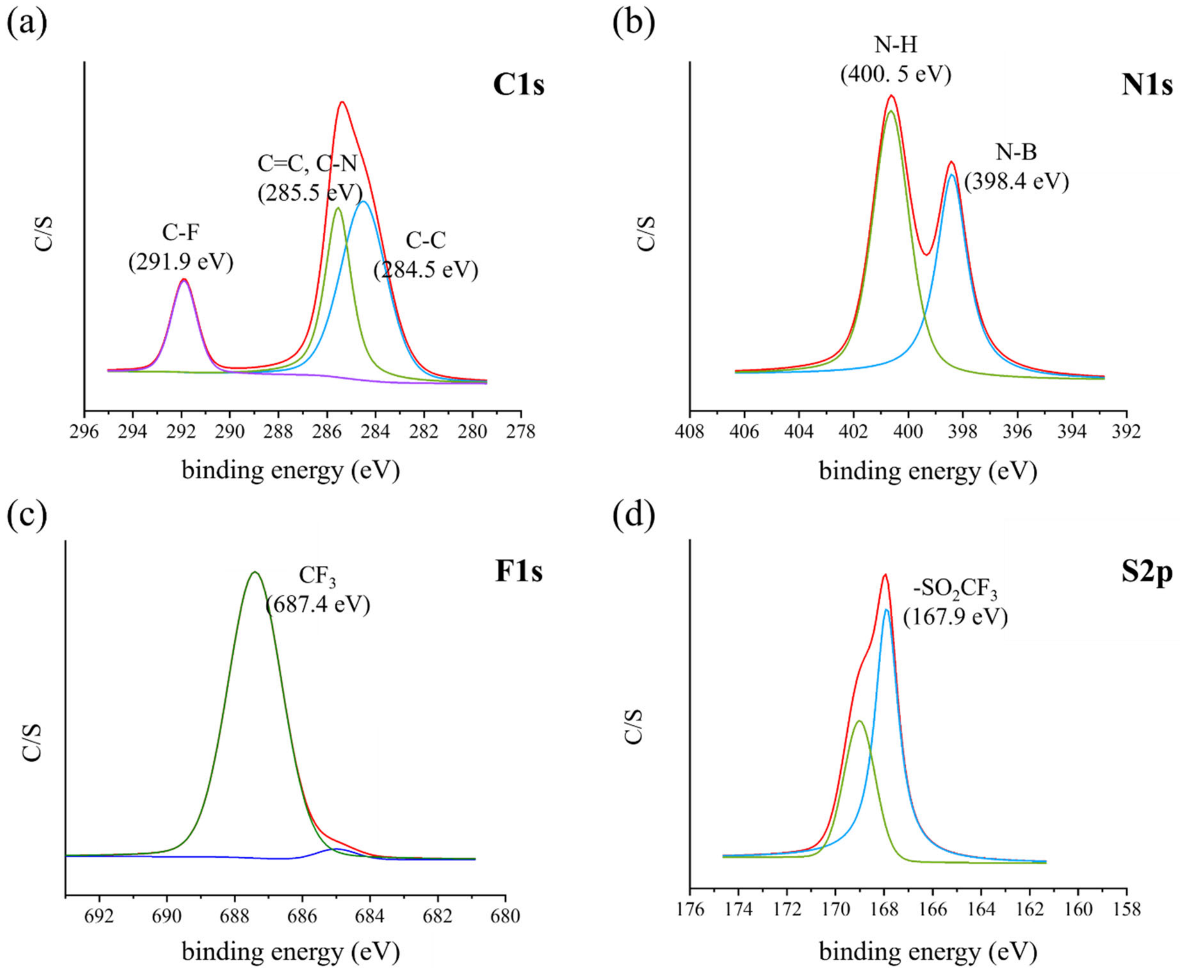

Figure S11. Post-cycled Pt surface characterization with x-ray photoelectron spectroscopy (XPS). (a) C1s, (b) N1s, (c) F1s, (d) S2p spectra of [(1-a-pyrr)$N_{111}BH_2$]TFSI.

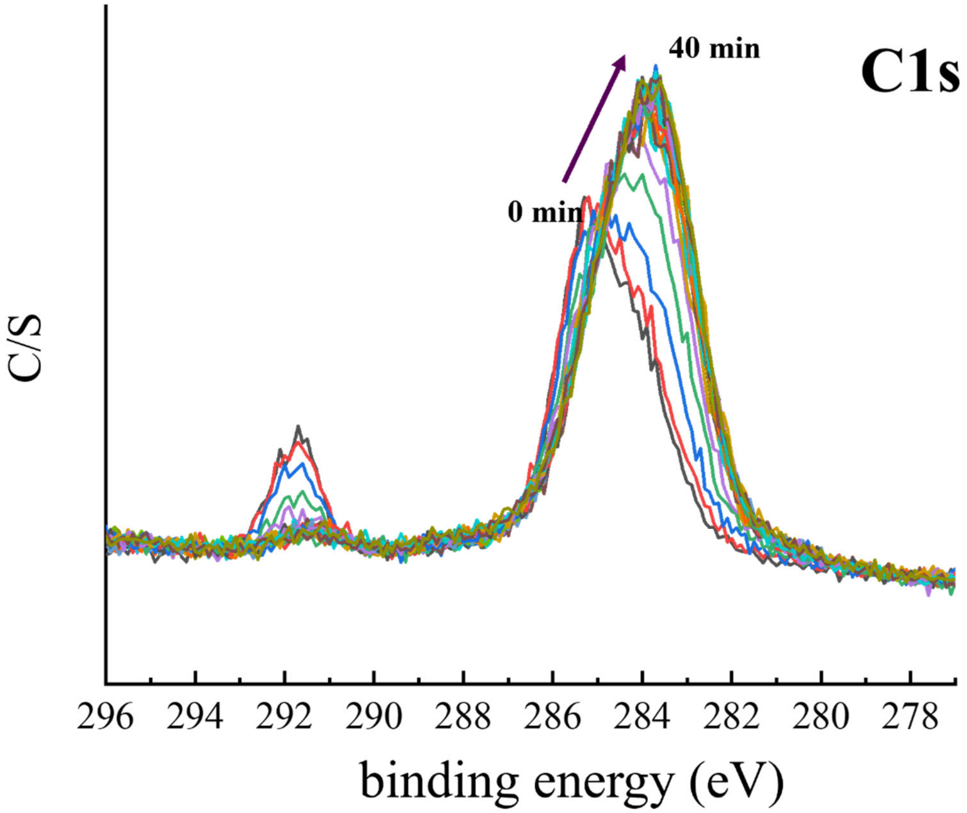

Figure S12. Electrode surface characterization with x-ray photoelectron spectroscopy (XPS). C1s spectra at different C60 sputtering times by sputter depth profiling (0 ~ 40 min).

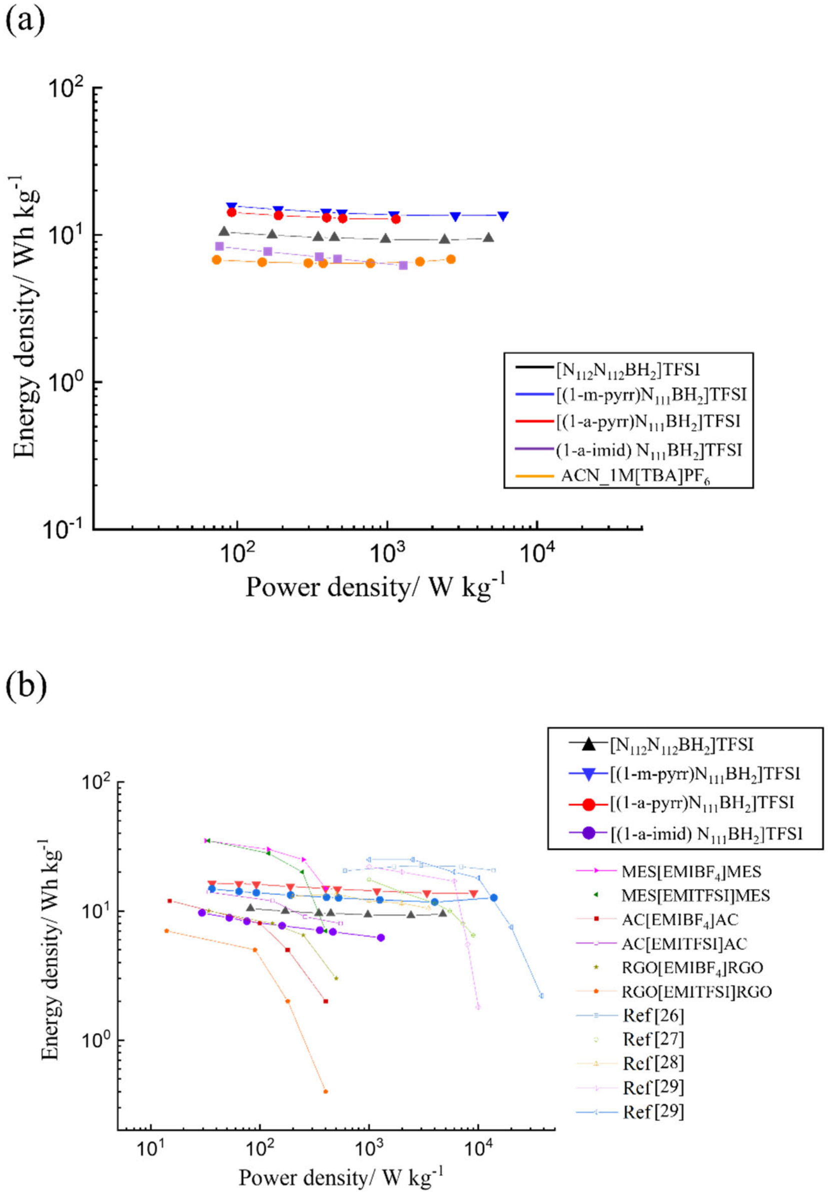

Figure S13. (a) Ragone plot depicting energy and power densities of the of boronium ionic liquids (BILs) electrolytes and organic electrolyte, ACN/1 M $[TBA]PF_6$. (b) Comparison of energy and power densities of each BILs against other carbon-based supercapacitors assembled with ionic liquids[24] and organic solvent electrolytes.[25]

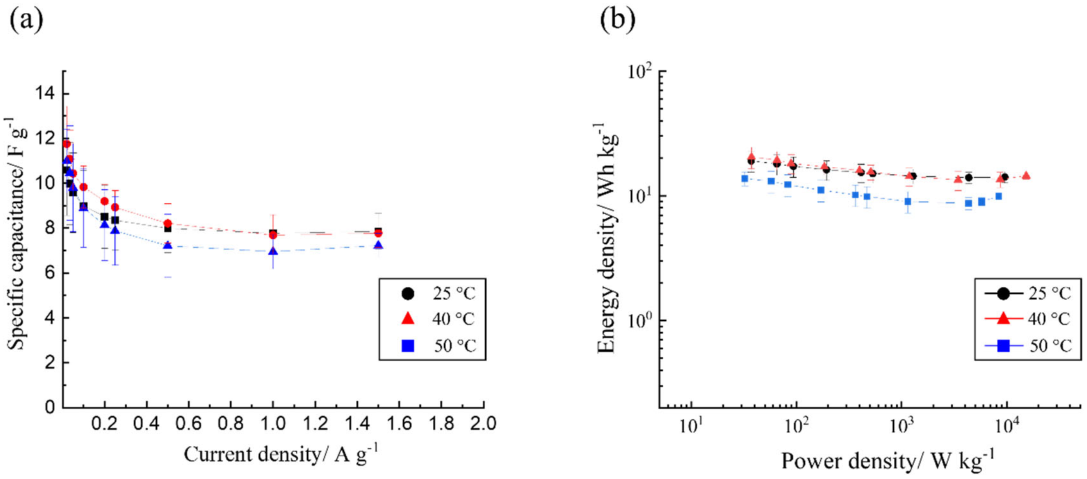

Figure S14. (a) Specific capacitance and (b) Ragone plot depicting energy and power densities of [(1-a-pyrr)$N_{111}BH_2$]TFSI at different temperatures from 25 °C to 50 °C.

REFERENCES

1. Moore, R. C.; White Jr, S. S.; Kelly, H. C.; Denton, D. L.; Shore, S. G., Borane addition compounds of bases. In *Inorg. Synth.*, Parry, R. W., Ed. 1970; Vol. XII, pp 109-115.
2. Garrett, J. M.; Ryschkewitsch, G. E.; Senor, L. E.; Carter, J. C., (4-Methylpyridine)(Trimethylamine)- Dihydroboron(III) Cation Salts. In *Inorg. Synth.*, 1970; pp 132-135.
3. Stachurski, C. D.; Cho, W.; Kinnaman, C. M.; Zeller, M.; Davis, J. H.; Larm, N. E.; Trulove, P. C.; Durkin, D. P., Evaluating Allyl and Vinyl-Modified Boronium Ionic Liquids for Energy Storage Applications. *J. Electrochem. Soc.* **2024,** *171* (12), 126506.
4. Krause, L.; Herbst-Irmer, R.; Sheldrick, G. M.; Stalke, D., Comparison of silver and molybdenum microfocus X-ray sources for single-crystal structure determination. *J. Appl. Crystallogr.* **2015,** *48* (1), 3-10.
5. Sheldrick, G. M., A short history of SHELX. *Acta Crystallogr.* **2008,** *64* (1), 112-122.
6. Sheldrick, G. M., SHELXT–Integrated space-group and crystal-structure determination. *J. Appl. Crystallogr.* **2015,** *71* (1), 3-8.
7. Sheldrick, G. M., Crystal structure refinement with SHELXL. *Cryst. Struct. Commun.* **2015,** *71* (1), 3-8.
8. Hübschle, C. B.; Sheldrick, G. M.; Dittrich, B., ShelXle: a Qt graphical user interface for SHELXL. *Applied Crystallography* **2011,** *44* (6), 1281-1284.
9. Zhou, D.; Wang, H.; Mao, N.; Chen, Y.; Zhou, Y.; Yin, T.; Xie, H.; Liu, W.; Chen, S.; Wang, X., High energy supercapacitors based on interconnected porous carbon nanosheets with ionic liquid electrolyte. *Micropor. Mesopor. Mat.* **2017,** *241*, 202-209.
10. Sun, G.; Li, K.; Xie, L.; Wang, J.; Li, Y., Preparation of mesoporous carbon spheres with a bimodal pore size distribution and its application for electrochemical double layer capacitors based on ionic liquid as the electrolyte. *Micropor. Mesopor. Mat.* **2012,** *151*, 282-286.
11. Fuertes, A. B.; Sevilla, M., High-surface area carbons from renewable sources with a bimodal micro-mesoporosity for high-performance ionic liquid-based supercapacitors. *Carbon* **2015,** *94*, 41-52.
12. Tran, C.; Lawrence, D.; Richey, F. W.; Dillard, C.; Elabd, Y. A.; Kalra, V., Binder-free three-dimensional high energy density electrodes for ionic-liquid supercapacitors. *Chem. Commun.* **2015,** *51* (72), 13760-13763.
13. Li, Z.; Liu, J.; Jiang, K.; Thundat, T., Carbonized nanocellulose sustainably boosts the performance of activated carbon in ionic liquid supercapacitors. *Nano Energy* **2016,** *25*, 161-169.
14. Sasi, R.; Sarojam, S.; Devaki, S. J., High performing biobased ionic liquid crystal electrolytes for supercapacitors. *ACS Sustain. Chem. Eng.* **2016,** *4* (6), 3535-3543.
15. Zhang, X.; Wang, L.; Peng, J.; Cao, P.; Cai, X.; Li, J.; Zhai, M., A flexible ionic liquid gelled PVA-Li2SO4 polymer electrolyte for semi-solid-state supercapacitors. *Adv. Mater. Interfaces* **2015,** *2* (15), 1500267.
16. Song, Z.; Li, L.; Zhu, D.; Miao, L.; Duan, H.; Wang, Z.; Xiong, W.; Lv, Y.; Liu, M.; Gan, L., Synergistic design of a N, O co-doped honeycomb carbon electrode and an ionogel electrolyte enabling all-solid-state supercapacitors with an ultrahigh energy density. *J. Mater. Chem. A* **2019,** *7* (2), 816-826.
17. Deng, X.; Li, J.; Zhu, S.; Ma, L.; Zhao, N., Boosting the capacitive storage performance of MOF-derived carbon frameworks via structural modulation for supercapacitors. *Energy Storage Mater.* **2019,** *23*, 491-498.

18. Tian, W.; Gao, Q.; Tan, Y.; Li, Z., Unusual interconnected graphitized carbon nanosheets as the electrode of high-rate ionic liquid-based supercapacitor. *Carbon* **2017,** *119*, 287-295.
19. Pereira, N. d. M.; Trigueiro, J. P. C.; Monteiro, I. d. F.; Montoro, L. A.; Silva, G. G., Graphene oxide–ionic liquid composite electrolytes for safe and high-performance supercapacitors. *Electrochim. Acta.* **2018,** *259*, 783-792.
20. Chaudoy, V.; Van, F. T.; Deschamps, M.; Ghamouss, F., Ionic liquids in a poly ethylene oxide cross-linked gel polymer as an electrolyte for electrical double layer capacitor. *J. Power Sources* **2017,** *342*, 872-878.
21. Tiruye, G. A.; Muñoz-Torrero, D.; Palma, J.; Anderson, M.; Marcilla, R., Performance of solid state supercapacitors based on polymer electrolytes containing different ionic liquids. *J. Power Sources* **2016,** *326*, 560-568.
22. Pandey, G. P.; Liu, T.; Hancock, C.; Li, Y.; Sun, X. S.; Li, J., Thermostable gel polymer electrolyte based on succinonitrile and ionic liquid for high-performance solid-state supercapacitors. *J. Power Sources* **2016,** *328*, 510-519.

23. Lindström, H.; Södergren, S.; Solbrand, A.; Rensmo, H.; Hjelm, J.; Hagfeldt, A.; Lindquist, S.-E. Li+ ion insertion in TiO2 (anatase). 2. Voltammetry on nanoporous films. *J. Phys. Chem. B* **1997**, *101* (39), 7717-7722. Ko, J. S.; Lai, C.-H.; Long, J. W.; Rolison, D. R.; Dunn, B.; Nelson Weker, J. Differentiating double-layer, psuedocapacitance, and battery-like mechanisms by analyzing impedance measurements in three dimensions. *ACS Appl. Mater. Interfaces.* **2020**, *12* (12), 14071-14078.
24. Ortega, P. F.; Santos Jr, G. A. d.; Trigueiro, J. P.; Silva, G. G.; Quintanal, N.; Blanco, C.; Lavall, R. L.; Santamaría, R. Insights on the behavior of imidazolium ionic liquids as electrolytes in carbon-based supercapacitors: an applied electrochemical approach. *J. Phys. Chem. C.* **2020**, *124* (29), 15818-15830.
25. Qi, Y.; Bao, C.; Qiu, J.; Hu, Z.; Lu, S.; Yan, J.; Cen, K.; Bo, Z.; Yang, H. Electrolyte Formulation with Improved Ion Desolvation and Diffusion Kinetics, and Superior Anti-Corrosion Properties for Ultrawide-Temperature Supercapacitors (–70∼ 100° C). *Energy Storage Mater.* **2024**, *72*, 103782.

26. Qi, Y.; Bao, C.; Qiu, J.; Hu, Z.; Lu, S.; Yan, J.; Cen, K.; Bo, Z.; Yang, H., Electrolyte Formulation with Improved Ion Desolvation and Diffusion Kinetics, and Superior Anti-Corrosion Properties for Ultrawide-Temperature Supercapacitors (–70∼ 100° C). *Energy Storage Mater.* **2024,** *72*, 103782.
27. Wu, D.; Xu, L. H.; Feng, H. J.; Zhu, Y. W.; Chen, X. Y.; Cui, P., Design and theoretical study of novel deep eutectic solvents: The effects of bromine and chloride anions on solvation structure and supercapacitor performance. *J. Power Sources* **2021,** *492*, 229634.
28. Suo, L.; Borodin, O.; Gao, T.; Olguin, M.; Ho, J.; Fan, X.; Luo, C.; Wang, C.; Xu, K., “Water-in-salt” electrolyte enables high-voltage aqueous lithium-ion chemistries. Science 2015, 350 (6263), 938-943.

29. Zhang, J.; Yang, H.; Huang, Z.; Zhang, H.; Lu, X.; Yang, J.; Cen, K., Bo, Z., Pore-structure regulation and heteroatom doping of activated carbon for supercapacitors with excellent rate performance and power density. Waste Dispos. Sustain. Energy 2023, 5, 417-426.
